# Scalable synthesis of 5,11-diethynylated indeno[1,2-*b*]fluorene-6,12-diones and exploration of their solid state packing

**DOI:** 10.3762/bjoc.10.219

**Published:** 2014-09-05

**Authors:** Bradley D Rose, Peter J Santa Maria, Aaron G Fix, Chris L Vonnegut, Lev N Zakharov, Sean R Parkin, Michael M Haley

**Affiliations:** 1 Department of Chemistry & Biochemistry and the Materials Science Institute, University of Oregon, Eugene, Oregon 97403-1253, USA; 2 CAMCOR, University of Oregon, 1443 East 13th Avenue, Eugene, Oregon 97403, USA; 3 Department of Chemistry, University of Kentucky, Lexington, Kentucky 40506-0055, USA

**Keywords:** crystal packing, electron accepting, indenofluorenes, organic electronics, polycyclic conjugated hydrocarbons

## Abstract

We report a new synthetic route to 5,11-disubstituted indeno[1,2-*b*]fluorene-6,12-diones that is amenable to larger scale reactions, allowing for the preparation of gram amounts of material. With this new methodology, we explored the effects on crystal packing morphology for the indeno[1,2-*b*]fluorene-6,12-diones by varying the substituents on the silylethynyl groups.

## Introduction

Polycyclic conjugated hydrocarbons (PCHs) have been studied extensively due to the wide variety of physical properties that can be accessed by appropriate manipulation or “tuning” of a molecular scaffold (e.g., installation of donor/acceptor groups, inclusion of heteroatoms, etc.) [1–3]. Recently there has been resurging interest in PCHs for use as active materials in organic electronic devices. Some popular examples of devices undergoing extensive exploration are organic field effect transistors (OFET) [4–5], organic photovoltaics (OPV) [6], and organic light emitting devices (OLED) [7]. For such devices to operate properly, these must include materials that conduct holes (electron donating) and conduct electrons (electron accepting) [8]. While there are many systems that display high hole mobilities, there are far fewer that exhibit high electron mobilities.

**Figure 1:**
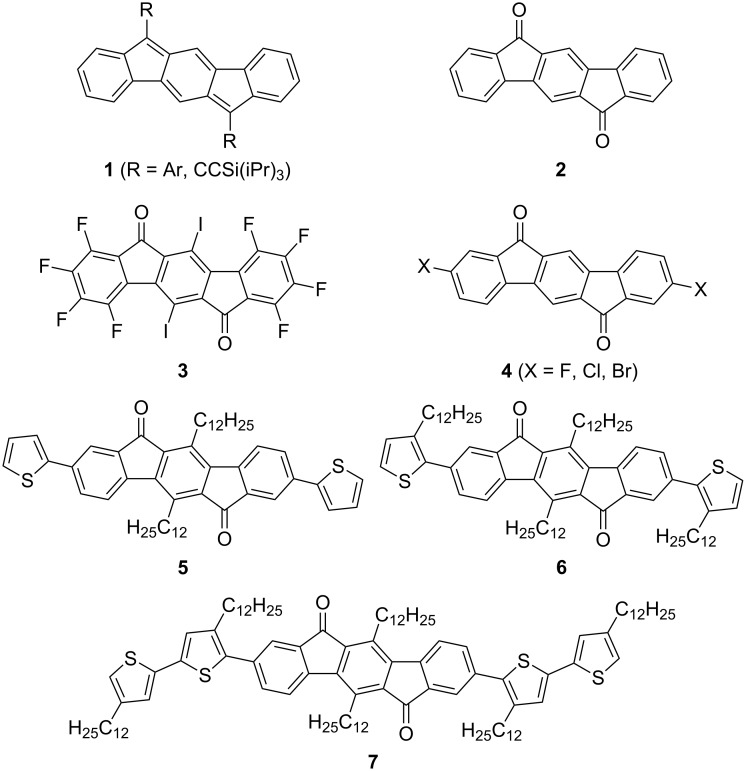
Previously reported indeno[1,2-*b*]fluorenes and related indeno[1,2-*b*]fluorene-6,12-diones.

Our laboratory has been exploring a new class of PCHs based on the five structural isomers of indenofluorene [9]. In particular, the indeno[1,2-*b*]fluorene (IF, **1**, Figure 1) skeleton is similar to linear oligoacenes, with the notable exception that the molecule contains two five-membered carbocycles. This modest alteration imparts an inherent propensity of the IF scaffold to be electron accepting [10–11]. A simple explanation for the high electron affinity of the IF is that to make all five rings formally aromatic two electrons must be added to the system, effectively creating two cyclopentadiene anions [12]. The result of the IFs high electron affinity is nearly balanced ambipolar charge transport in OFETs [11,13].

The synthetic precursors to **1**, the indeno[1,2-*b*]fluorene-6,12-diones (IF-diones, **2**, Figure 1) have also been explored as an active layer in OFETs. The first reported IF-dione OFET utilized **3** – while the solid-state structure of **3** showed several sub-van der Waals contact distances, the n-type mobility of the OFET was very low (2 × 10^−5^ cm^2^ V^−1^ s^−1^) [14]. On the other hand, an OFET utilizing **4** (X = F) had measured electron mobilities of 0.17 cm^2^ V^−1^ s^−1^, and its X-ray crystal structure revealed 1-dimensional π-stacking with a close interplanar distance of 3.30 Å [15]. Due to the inherent insolubility of compounds **3** and **4**, however, they needed to be vapor deposited under vacuum. More recently IF-diones **5**–**7** were reported (along with polymeric and other derivatives) with **5** and **6** exhibiting both n- and p-channel behavior in OFETs [16]. Notably, **6** showed balanced hole and electron mobilities when vapor or solution processed. Molecule/polymer solubility is desirable because it offers the benefit of being solution processable, which could allow for the inexpensive large area printing of electronic devices.

We report herein the preparation of a variety of diethynylated IF-diones **8a–j** that are readily soluble in common organic solvents, and the exploration of their packing in the solid-state by X-ray crystallography. The prototypical molecule that served as inspiration for our studies was pentacene, as it, along with numerous other acene derivatives, has been substituted with trialkylsilylethynyl groups of varying size to study the effect on the solid state packing in single crystals [17]. This was shown to have a large effect on the OFET performance as slight changes in the geometry can dramatically alter the intermolecular electronic coupling, which is what ultimately dictates performance of the device [18–19].

## Results and Discussion

**Scheme 1:**
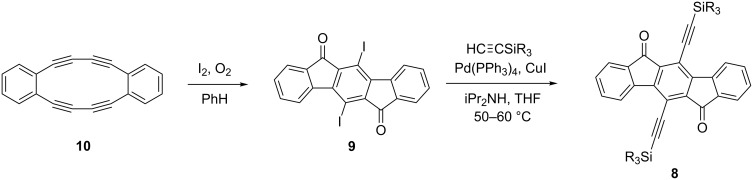
Transannular cyclization route to diethynyl-IF-diones **8**.

**Synthesis.** Our initial studies [20] toward **8** (Scheme 1) focused on the Sonogashira cross-coupling of known diiodo intermediate **9**, which was prepared by double transannular cyclization of **10** using elemental iodine under air [21]. Dehydrobenzo[12]annulene **10** in turn was synthesized via Glaser homocoupling of 1,2-diethynylbenzene [21–23]. While in theory this route permitted relatively easy access to diethynyldiones **8**, in practice it was fraught with problems: (1) the formation of **10** was very sensitive to the reaction conditions and thus typically gave low yields (approx. 10%) upon scale-up; (2) the reaction must be run in very dilute solution to minimize the formation of larger cyclooligomers as well as polymer; (3) pure **10** in the solid state is reported to be shock sensitive [22], a fact that we can readily reaffirm; and (4) the iodine atoms on **9** are quite labile as we often observed formation of elemental iodine if solutions of **9** were exposed to heat or sunlight. If we wanted to obtain quantities of diones **8** beyond 20–30 milligrams at a time, we had to overcome the synthetic roadblock that Scheme 1 represented.

**Scheme 2:**
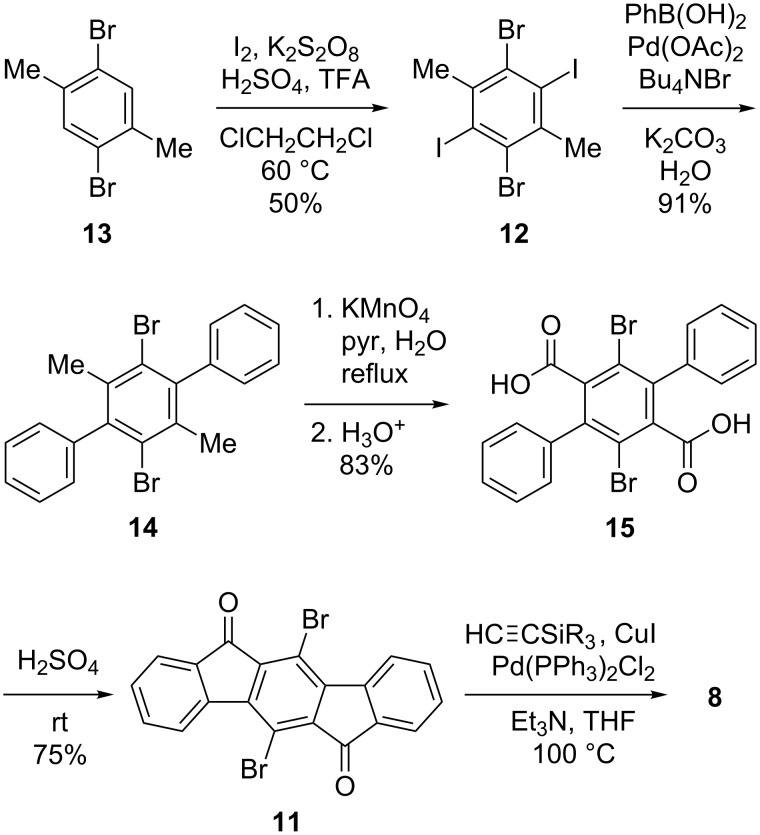
Suzuki/Friedel-Crafts route to diethynyl-IF-diones **8**.

The improved synthetic route to **8** arises from a retrosynthetic analysis of the current method to prepare IF derivatives [9–11,13]. The needed modification must include halogens at the 5 and 11 positions for subsequent functionalization, such as the more robust bromines in **11**, yet avoid annulene transannular synthesis [24]. Instead, the route we chose involved key precursor **12**, which surprisingly is an unknown compound. Starting with commercially available 2,5-dibromo-*p*-xylene (**13**) (Scheme 2), iodination using the method reported by Kitamura gave tetrahalide **12** in good yield on >10 g scale [25]. Suzuki cross-coupling with **12** furnished *p*-terphenyl **14**, followed by oxidation of the methyl groups to produce diacid **15**. Intramolecular Friedel–Crafts acylation then afforded 5,11-dibromo-IF-dione **11**. The yields for the Sonogashira cross-coupling of a variety of trialkylsilylacetylenes to either **9** or **11** were modest to very good (Table 1) but were not optimized.

**Table 1:** Diethynyl-IF-diones synthesized and yields for Sonogashira cross-coupling.

	trialkylsilyl group			isolated yield	
	R^1^	R^2^	R^3^	from **9**	from **11**

**8a**	Me	Me	Me	40	–
**8b**	Et	Et	Et	8	27
**8c**	*n-*Pr	*n-*Pr	*n-*Pr	–	72
**8d**	iPr	iPr	iPr	61	48
**8e**	iBu	iBu	iBu	7	–
**8f**	Ph	Ph	Ph	15	–
**8g**	Me	Me	CF_3_(CH_2_)_2_	–	30
**8h**	Me	Me	iBu	–	35
**8i**	Me	Me	*t-*Bu	17	–
**8j**	Me	Me	Ph	–	24

**Figure 2:**
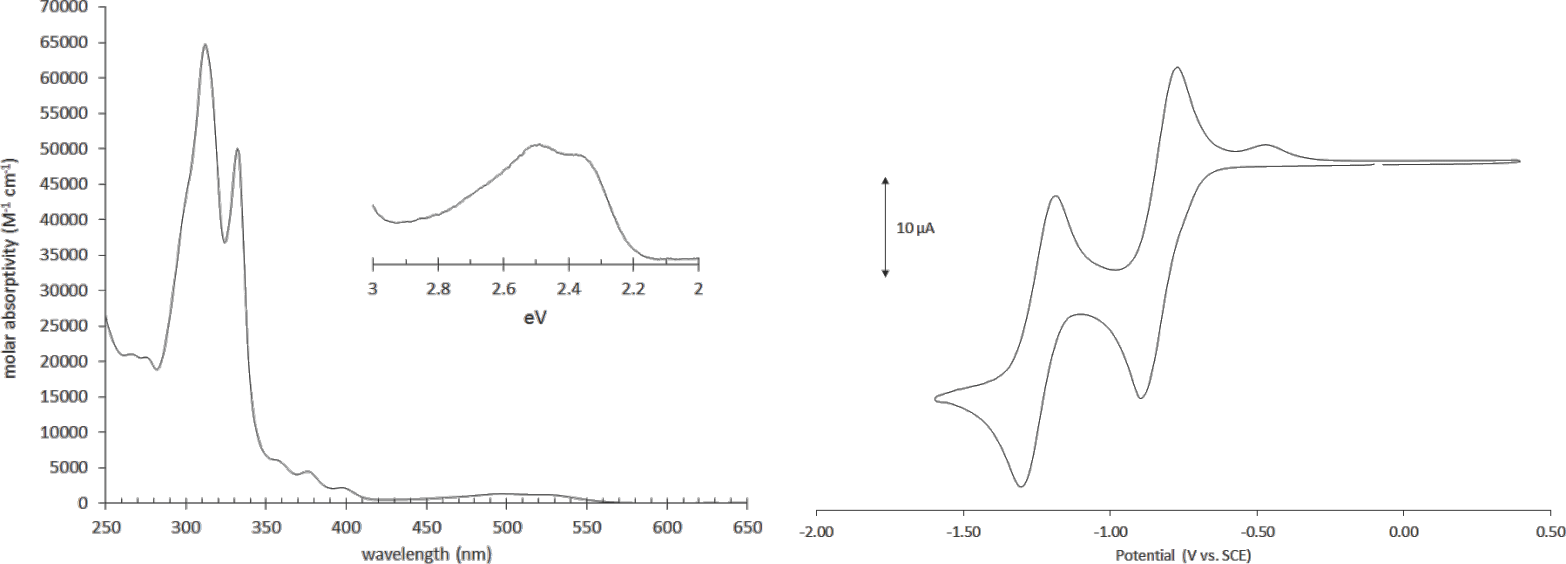
UV–vis spectrum (left) and cyclic voltammogram (right) of dione **8c**.

**Optical and electronic properties.** Shown in Figure 2 are the UV–vis spectrum and the cyclic voltammogram of **8c**, data that are representative of all the 5,11-diethynyl-IF-diones. As anticipated, altering the trialkylsilyl group has very little effect on the optoelectronic properties of the conjugated scaffold (Table 2). All molecules have two strong absorptions around 310 and 330 nm, with a much weaker, broad absorption in the 450–550 nm range. Electrochemistry shows two reversible reductions with potentials of −0.78 to −0.84 V for the first reduction and −1.18 to −1.26 V for the second reduction. The small differences in the absorbance and cyclic voltammetry essentially fall within experimental error.

**Table 2:** Electrochemical and optical data for ID-diones **8a–j**.

	electrochemical^a^				optical	
compd	*E*_red_^1^ (V)	*E*_red_^2^ (V)	*E*_LUMO_ (eV)^b^	*E*_HOMO_ (eV)^c^	λ_abs_ (nm)	gap (eV)^d^

**8a**	−0.80	−1.21	−3.89	−6.26	310, 330, 524	2.37
**8b**	−0.79	−1.23	−3.90	−6.28	311, 332, 522	2.38
**8c**	−0.83	−1.24	−3.86	−6.23	312, 332, 524	2.37
**8d**	−0.82	−1.24	−3.87	−6.23	313, 333, 525	2.36
**8e**	−0.84	−1.26	−3.85	−6.22	314, 333, 524	2.37
**8f**	−0.78	−1.18	−3.90	−6.29	312, 333, 520	2.39
**8g**	−0.77	−1.18	−3.87	−6.27	308, 330, 516	2.40
**8h**	−0.78	−1.21	−3.86	−6.24	310, 331, 521	2.38
**8i**	−0.81	−1.25	−3.87	−6.23	312, 331, 526	2.36
**8j**	−0.78	−1.20	−3.86	−6.24	310, 331, 521	2.38

^a^CV recorded using 1–5 mM of analyte in 0.1 M solution of either Bu_4_NOTf (**8a, 8b**, **8d–f**, **8i)** or Bu_4_NBF_4_ (**8c**, **8g**, **8h**, **8j**) in HPLC-grade CH_2_Cl_2_. Values reported as the half-wave potential (vs SCE) using the Fc/Fc^+^ couple (0.46 V) as an internal standard. See Supporting Information File 1 for details. ^b^LUMO energy levels were approximated using SCE = −4.68 eV vs vacuum [26]. ^c^Estimated by subtracting the optical bandgap from *E*_LUMO_. ^d^Estimated from the λ_max_ of the lowest energy UV–vis peak.

**Figure 3:**
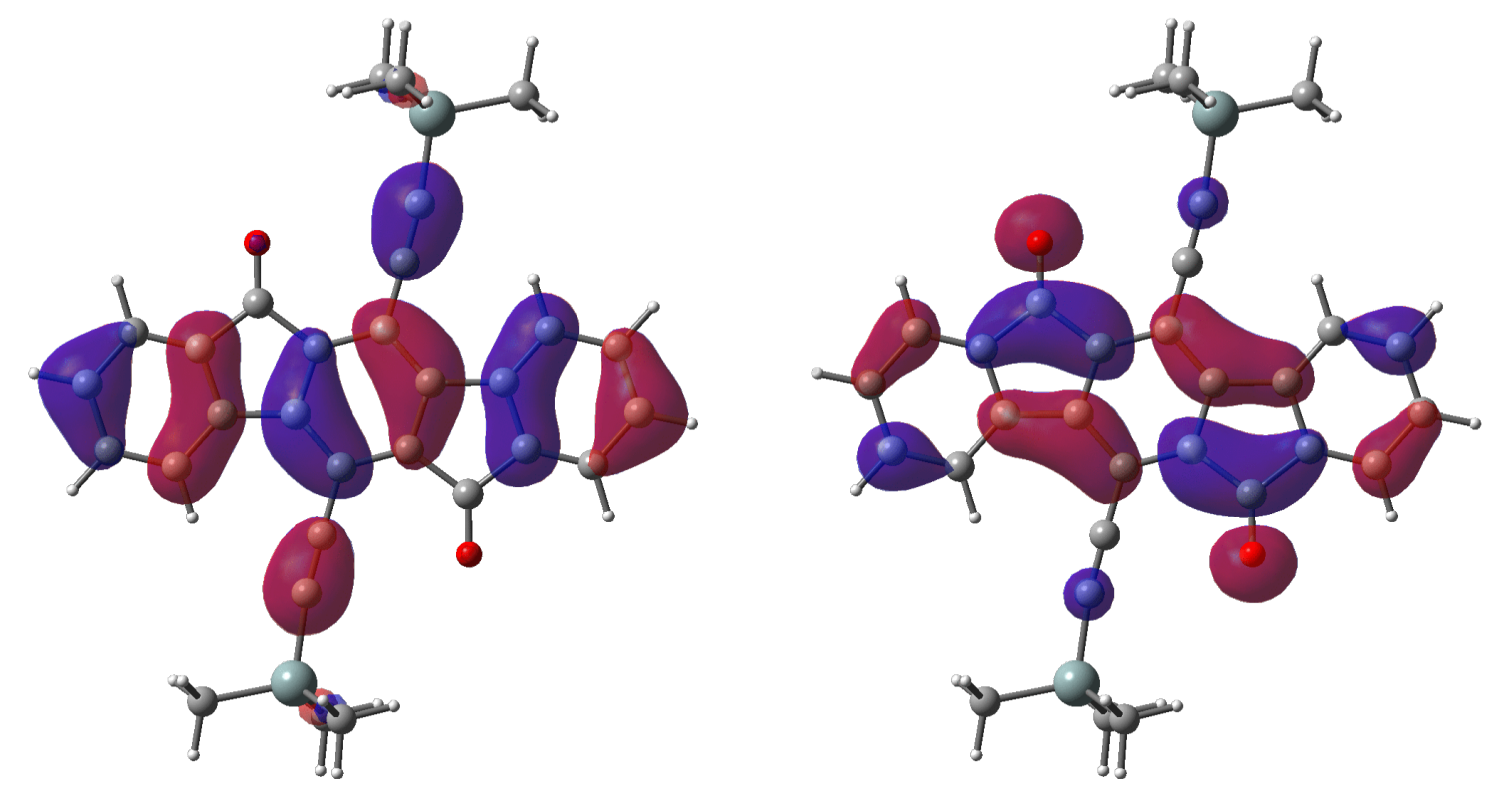
Kohn–Sham HOMO (left) and LUMO (right) plots of **8a**.

Interestingly these compounds have a low energy S_0_→S_1_ transition at ca. 500–525 nm which has previously and incorrectly been described as an n→π* transition [16,20]; however, TD-DFT calculations predict this to be π→π* (Figure 3) [27–28]. The n→π* transition was calculated to have a slightly higher energy transition with an oscillator strength of 0; thus, it should not be visible in the UV–vis spectrum (Table 3). To see if this was a computational artifact, the same calculations were performed for fluorenone and benzophenone, where it has previously been established that the S_0_→S_1_ transition corresponds to π→π* and n→π*, respectively [29–30]. The calculations correctly predict the ordering of the states for fluorenone and benzophenone. To validate this experimentally, UV–vis spectra were gathered in solvents of differing polarity. We anticipated that if the S_0_→S_1_ transition corresponds to a n→π* transition, the energy separating the S_0_ and S_1_ states would be measurably different in polar solvents when compared to non-polar solvents, thus leading to an energy shift of this transition. Likewise if the S_0_→S_1_ transition was a π→π* there should be essentially no change in the transition energy when changing solvent polarity. We found that the shift in the spectrum upon changing solvent polarity in going from *n*-hexane to acetone was 0.02 eV, supporting our hypothesis that the 450–550 nm absorption is indeed a π→π* transition as indicated by calculations.

**Table 3:** Calculated transitions for **8a**, showing only the main contribution to each excitation. Calculated using TD-B3LYP/6-311+G(d,p)//B3LYP/6-31G(d).

transition	molecular orbitals	contribution to excitation, %	oscillator strength	energy, eV (nm)

S_0_→S_1_	HOMO→LUMO	97	0.0295	2.35 (527)
S_0_→S_2_	HOMO-2→LUMO	89	0.0000	2.71 (458)
S_0_→S_3_	HOMO-3→LUMO	80	0.0000	2.86 (434)
S_0_→S_4_	HOMO-1→LUMO	88	0.0486	2.95 (420)

**X-ray crystallography.** We explored the solid-state packing geometries resulting from altering the substitution on the silyl groups. The high crystallinity of the majority of the compounds examined permitted facile growth of large single crystals, of approximately several millimeters, from hexanes solution. Single crystals for **8g** and **8j** were grown from chlorobenzene due to the low solubility and crystallinity of these compounds. The molecular structures were then elucidated using x-ray diffraction.

**Figure 4:**
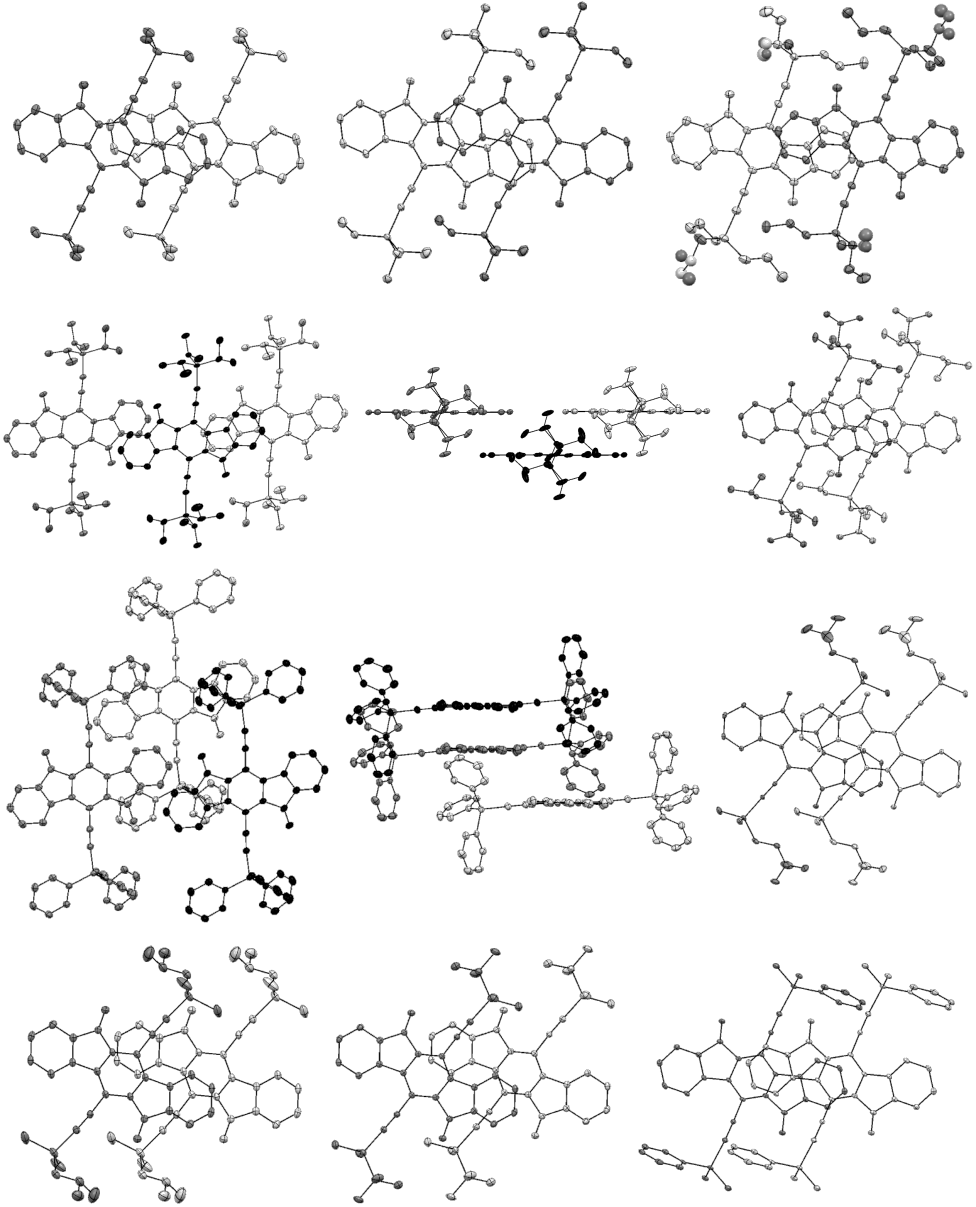
Views perpendicular to the average plane of the π stack. 1^st^ row left to right – **8a**, **8b**, **8c**; 2^nd^ row – **8d**, **8d**, **8e**; 3^rd^ row – **8f**, **8f**, **8g**; 4^th^ row **8h**, **8i**, **8j**. Hydrogen atoms omitted for clarity; ellipsoids drawn at the 30% probability; individual molecules were colored the same to identify overlap easier.

The ten compounds **8a**–**j** exhibit several different packing motifs (Figure 4). In a broad sense, the progression in packing follows a trend with the volume of the substituted silyl groups [31–32]. Segregation of the IF-dione backbone and the solubilizing groups, and π-stacking is observed in all but the largest (**8f**, SiPh_3_), but there are significant differences in the nature of the π-stacking of the other nine compounds. The smallest (**8a**, SiMe_3_, vol. ~130 Å^3^) is monoclinic, *P*2_1_/c, with a unit cell elongated along *b*. The molecules form 1-D π-stacks (interplanar spacing 3.446(5) Å) parallel to *a*, with adjacent stacks having the opposite tilt by virtue of the *c*-glide, leading to a herringbone motif in which inter-stack interactions are solely between the SiMe_3_ groups. This is the only structure of this type in the group of ten compounds. The next largest (**8g**, SiMe_2_PrF_3_, vol. ~198 Å^3^) is orthorhombic, *P*ca2_1_, with a squat unit cell (short *b* axis). Molecules form 1-D π-stacks parallel to the *b*-axis (interplanar spacing 3.358(3) Å), while adjacent stacks interact via C-H···π contacts to form herringbone-patterned layers (interstack molecular tilt 66.85(2)°) in the *ab* plane, separated by layers of fluorinated ‘grease’. Compounds **8b**, **8i** and **8h**, which have essentially the same volume solubilizing groups (~204 Å^3^), exhibit very similar overall packing motifs. They are monoclinic, *P*2_1_/c, with squat unit cells (short *b* axis), and form 1-D π-stacks parallel to *b* (interplanar spacings of 3.454(2) Å, 3.340(2) Å and 3.385(3) Å, respectively, for **8b**, **8i** and **8h**). Adjacent stacks along *c* interact via C–H···π-contacts, and are related by the *c*-glide operation to form the common herringbone motif (interstack molecular tilts of 64.88(2)°, 64.45(2)° and 68.99(3)°, respectively, for **8b**, **8i** and **8h**). These *bc* layers are separated by layers of trialkylsilyl groups. Compound **8j** has a slightly larger (~210 Å^3^) solubilizing group, but is triclinic, 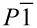
*.* The difference in crystal symmetry compared to **8b**, **8i**, **8h** (and to **8g**), however, belies the similarities. The unit cell is a squat skewed box (short *a*-axis, though easily transformed to a short *b* setting that makes the similarity to **8b**, **8i**, **8h** clearer). There are two half molecules per asymmetric unit, each sitting on inversion centers. Crystallographically identical molecules form 1-D π-stacks along the short *a*-axis (*cf* short *b*-axis in **8b**, **8i**, **8h**, **8g**), leading to two independent molecular π-stacks, which have slightly different interlayer spacings (3.385(2) Å and 3.397(2) Å). Between adjacent stacks, the crystallographically independent molecules are tilted relative to each other by 66.88(2)°, giving a variant of the common herringbone motif, despite the lack of a crystallographic glide plane. In addition to the IF-dione π-stacking, inversion-related phenyl groups on the solubilizing groups are paired by π–π interactions. The stacking in compounds **8c** and **8d** (Si(*n-*Pr)_3_ and Si(iPr)_3_, vol. ~278 Å^3^) are similar at first glance. In **8c**, each end of the IF-dione backbone overlaps by different amounts with adjacent molecules (interplanar spacings of 3.422(3) Å and 3.446(4) Å) to form a 2-D π-stacked brickwork motif. In **8d**, the superficially similar π-stacking motif is better described as a 1-D π-stack. There is significant overlap between adjacent molecules on only one side (interlayer spacing = 3.404(3) Å), while on the other side, any 'overlap' amounts to only the mutual superposition of C10 over the C5–C10 bond of the neighboring molecule. Moreover, a rudimentary superposition of the LUMO plots (Figure 3) for the relative positions of this molecular pair arrangement suggests no favorable orbital interactions. In **8e** (Si(iBu)_3_, vol. ~353 Å^3^), the structure is monoclinic, *P*2_1_/n. The IF-dione molecules form 1-D π-stacks (interplanar spacing = 3.394(2) Å) that are completely segregated from neighboring stacks by the bulky Si(iBu)_3_ groups. The largest group, SiPh_3_ (vol. ~372 Å^3^) in **8f**, effectively suppresses overlap of the IF-dione backbones between adjacent molecules.

**Figure 5:**
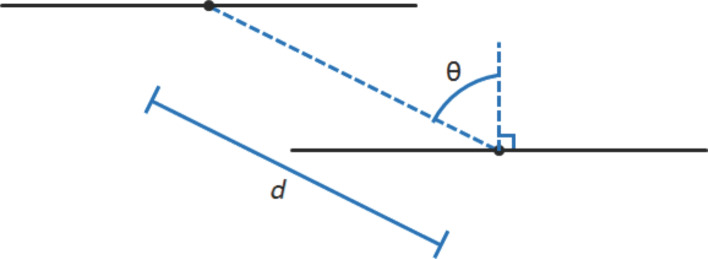
Schematic of the parameters used for comparing X-ray crystal structures, view is parallel to the molecular plane. Black lines represent the molecules with circles denoting the centroid.

There are three distinct substitution patterns in the array of IF-diones synthesized – (1) the three groups are *n*-alkyl chains with symmetry (three mirror symmetry planes about the silicon), (2) bulky alkyl groups with symmetry, and (3) dimethyl-substituted possessing only one mirror plane of symmetry about the silicon. When looking further for trends, we compared two parameters to see if any of them ultimately yielded packing motifs with close contacts between the carbons of the conjugated system. One parameter examined is the distance between the centroid of the planar system and its next nearest neighbor along the one-dimensional π stack. The other parameter is the angle between the centroids of the nearest neighbor molecules and the normal to the plane of a molecule; thus, a system with maximum overlap would have θ = 0°, while θ = 90° would result in no π orbital overlap. Using these two parameters a crude model for examining the possible intermolecular electronic coupling can be developed, which is pictorially represented in Figure 5.

Looking at Table 4 the only visible trend in the series appears to be within the *n*-alkyl symmetric. The *n*-alkyl symmetric series has both the *d* and θ follow the trend of the alkyl group’s radius. When examining symmetric and asymmetric series there is not an easily interpretable trend between the radii and *d* or θ. Pictorially the overlap between the molecular planes is clearly visible in Figure 5. For the *n*-alkyl symmetric series visual inspection reveals that the overlap between the planes of the molecules is greatest for the smallest group, **8a**, and least for the largest group, **8c**. Yet again, there is no clear visual trend in the overlap between the symmetric and asymmetric series. Considering that derivatives of **8** are most likely an n-channel material in OFETs, the density and phase of the LUMO should be the most important since in a molecular orbital picture of charge transport these are the orbitals that the extra electron would occupy. The Kohn–Sham LUMO density in Figure 3 is predominately located on the indacene moiety and the oxygen. From this perspective the best candidates for n-channel OFET materials would have large overlap between the indacene moiety and oxygen. In **8a**–**j** the oxygen is pointing away from the indacene moiety in the neighboring molecules along the stack and there is little to no overlap between the indacene moieties.

**Table 4:** Sizes of trisubstituted-silylethynyl groups in **8**.

				intermolecular close contacts			
		radius (Å)^a^	radius (Å)^b^	contact	distance (Å)	*d*^c^	θ^d^

*n*-alkyl symmetric	**8a**	2.69	–	–	–	6.366	57.2
	**8b**	4.06	–	–	–	6.439	57.5
	**8c**	5.26	–	–	–	8.239	65.4
symmetric	**8a**	2.69	–	–	–	6.366	57.2
	**8b**	4.06	–	–	–	6.439	57.5
	**8d**	4.09	–	–	–	9.550, 7.419^e^	69.6, 62.7^e^
	**8c**	5.26	–	–	–	8.239	65.4
	**8e**	5.28	–	C9···C2 C6···C3 C6···C6	3.407 3.562 3.524	6.134	56.4
	**8f**	5.89	–	C10···C10 C17···C4	3.188 3.369	10.984	74.7
asymmetric	**8i**	2.73	3.99	C7···C3	3.348	6.356	58.0
	**8h**	2.68	5.26	C9···C2 C10···C3	3.437 3.409	5.976	55.3
	**8g**	2.67	5.74	C6···C10 C3···C17	3.361 3.361	6.096	56.5
	**8j**	2.68	5.94	–	–	6.292	57.3

^a^Si···X distance where X is the farthest atom from Si with the covalent radii of X added to the distance [30]. ^b^Radius for other axis of lower symmetry trialkylsilanes. ^c^Distance between the centroid of two molecules in the 1-D stack. ^d^Angle between the centroid of each of two molecules of the π stack and the normal to the average plane. ^e^There are two symmetrically independent 1-D stacks.

## Conclusion

We have described an improved synthetic route to 5,11-diethynyl-functionalized indeno[1,2-*b*]fluorene-6,12-diones that permits a scalable synthesis of larger amounts of material. We explored the solid state packing motifs that result from altering the bulkiness as well as directionality of the trialkylsilyl groups. Altering the substitution on the silyl group had little, if any, effect on the electronic properties of **8**, which are dominated by the conjugated core; however, there were marked differences in the solid-state packing of single crystals of these compounds. Unfortunately, from a zeroth order approximation none of the variants displayed promising intermolecular electronic coupling.

## Supporting Information

File 1 Experimental procedures, computational details and xyz coordinates, X-ray information including CCDC numbers and copies of ^1^H and ^13^C NMR spectra.
