# Supplementary material for: Scalable synthesis of 5,11-diethynylated indeno[1,2-b]fluorene-6,12-diones and exploration of their solid state packing
Source: Beilstein J Org Chem. 2014 Sep 5;10:2122–30. doi: 10.3762/bjoc.10.219 (PMC4168888; doi:10.3762/bjoc.10.219)
Supplement: File 1 — Experimental procedures, computational details and xyz coordinates, X-ray information including CCDC numbers and copies of 1H and 13C NMR spectra. [file Beilstein_J_Org_Chem-10-2122-s001.pdf]

Supporting Information

for

Scalable synthesis of 5,11-diethynylated indeno[1,2-*b*]fluorene-6,12-diones and exploration of their solid state packing

Bradley D. Rose[1], Peter J. Santa Maria[1], Aaron G. Fix[1], Chris L. Vonnegut[1], Lev N. Zakharov[2], Sean R. Parkin[3] and Michael M. Haley*[1]

Address: [1]Department of Chemistry & Biochemistry and the Materials Science Institute, University of Oregon, Eugene, Oregon 97403-1253, USA, [2]CAMCOR, University of Oregon, 1443 East 13th Avenue, Eugene, Oregon 97403, USA and [3]Department of Chemistry, University of Kentucky, Lexington, Kentucky 40506-0055, USA

Email: Michael M. Haley - haley@uoregon.edu

*Corresponding author

Experimental procedures, computational details and xyz coordinates, X-ray information including CCDC numbers and copies of ^{1}H and ^{13}C NMR spectra

Table of Contents — Page Number

Table of Contents	Page Number
Experimental details	S2
Electronic absorption spectra	S7
Electrochemical details	S8
Cyclic voltammmograms	S8
X-ray crystallography	S9
Computational details	S11
References	S15
Copies of ^{1}H and ^{13}C NMR spectra	S16

Experimental details

General methods. ^{1}H and ^{13}C NMR spectra were recorded on a Bruker Avance III HD 600 with Prodigy BBO multinuclear Cryoprobe (^{1}H: 599.98 MHz, ^{13}C: 150.88 MHz) or Varian Mercury 300 MHz (^{1}H: 300.09 MHz). Chemical shifts are reported in parts per million (ppm) relative to tetramethylsilane, which was referenced according to trace amounts of non-deuterated chloroform (7.26 ppm) or dimethylsulfoxide (2.5 ppm) for ^{1}H and $CDCl_3$ (77.23 ppm) and DMSO-d_6 (39.51 ppm) for ^{13}C. Absorption spectra were recorded on HP 8453 UV–vis spectrometer. Glassware was dried in an oven at 100 °C and cooled under a stream of inert gas before use. Dry THF was distilled from potassium metal using benzophenone as an indicator under a nitrogen atmosphere. Sorbent Technologies silica G TLC plate w/UV254 aluminum backed, 200μm was used for TLC. Chromatography was performed using silica gel, tech grade, 60 Å, 230-400 mesh from Sigma-Aldrich. All commercial reagents were used as received unless otherwise noted. 5,11-Diiodoindeno[1,2-b]fluorene-6,12-dione (**2**),[1] (triisobutylsilyl)acetylene,[2] and (triphenylsilyl)acetylene[3] were prepared according to literature procedures.

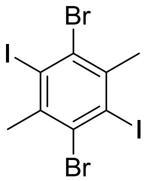

2,5-Dibromo-3,6-diiodo-*p*-xylene (12). 2,5-Dibromo-*p*-xylene (10 g, 37.9 mmol), $K_2S_2O_8$ (33.8 g, 125 mmol) and I_2 (31.7 g, 125 mmol) in CH_2Cl_2 (380 mL) were cooled in an ice bath and then a mixture of TFA (151 mL) and H_2SO_4 (6.8 mL) was added slowly. The reaction flaks was covered with Al foil and stirred at 60 °C. After 1 d an aliquot from the reaction was analyzed and if not complete, additional I_2 (6 g) and $K_2S_2O_8$ (6 g) were added. When complete the reaction was quenched by slowly pouring into cold H_2O (ca. 500 mL). The reaction was washed with H_2O several times and then the organic layer was reduced to half volume in vacuo. The resultant colored solid was collected and washed with acetone to yield **12** (9.77 g, 18.9 mmol,

50%) as a white solid. ^{1}H NMR (300 MHz, $CDCl_3$) δ 3.03 (s, 6H); ^{13}C NMR (151 MHz, $CDCl_3$): δ 142.45, 131.02, 110.63, 37.61; HRMS (TOF MS ESI+) $[M]^+$ calcd for $C_8H_6Br_2I_2^+$ 513.6926; found 513.6906.

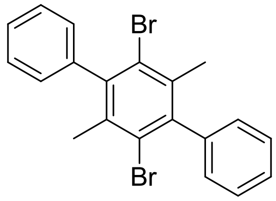

2,5-Dibromo-3,6-dimethyl-*p*-terphenyl (14). A flask was charged with diiodide **12** (3.68 g, 7.14 mmol), phenylboronic acid (2.61 g, 21.4 mmol), K_3PO_4 (5.61 g, 29 mmol) and $Pd(dppf)Cl_2•CH_2Cl_2$ (0.11 g, 0.14 mmol). Another flask was filled with dioxane (100 mL) and H_2O (27 mL). Both flasks were purged with N_2 or Ar for 45 min. The solvent was then transferred via cannula to the reaction flask and the mixture heated to 60 °C overnight. When complete, the cooled reaction was extracted with Et_2O and washed with H_2O, 10% HCl solution, H_2O and then dried over $MgSO_4$. The solvent was removed under reduced pressure and the white solid was used as collected (2.71 g, 6.52 mmol, 91%). Analytically pure crystals of **14** were obtained by recrystallization from Et_2O. ^{1}H NMR (600 MHz, $CDCl_3$) δ 7.47-7.52 (m, 4H), 7.40-7.44 (m, 2H), 7.20 (m, 4H), 2.20 (s, 6H); ^{13}C NMR (151 MHz, $CDCl_3$): δ 143.34, 142.56, 135.73, 129.19, 128.71, 127.70, 126.80, 23.49; HRMS (TOF MS ESI+) $[M]^+$ calcd for $C_{20}H_{17}Br_2^+$ 414.9697; found 414.9704.

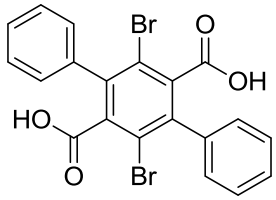

2,5-Dibromo-3,6-diphenylterephthalic acid (15). *p*-Terphenyl **14** (2.51 g, 6 mmol), $KMnO_4$ (12 g, 76 mmol), pyridine (150 mL) and H_2O (20 mL) were added to a flask equipped with a mechanical stirrer and condenser. The reaction was heated to reflux and four additional

portions of $KMnO_4$ (12 g each) were added every hour after reaching reflux. The mixture was refluxed overnight until the purple color of $KMnO_4$ was no longer visible. After cooling, the solid salts were collected by filtration and washed with of boiling 10% KOH solution. The combined solutions were then reduced to approximately half volume. After cooling to rt, the resulting solution was acidified carefully with conc. HCl and then cooled on ice. The precipitate was collected, washed with H_2O (ca. 100 mL) and dried overnight at 70 °C to give **15** (2.38 g, 5.0 mmol, 83%) as a white solid. Analytically pure samples were obtained by recrystallization from EtOH. ^{1}H NMR (600 MHz, DMSO-d_6) δ 13.62 (br s, 2H), 7.42-7.50 (m, 6H), 7.29-7.32 (m, 4H); ^{13}C NMR (151 MHz, DMSO-d_6): δ 166.73, 139.82, 139.39, 137.38, 129.39, 128.57, 128.15, 118.81; HRMS (TOF MS ESI+) $[M]^+$ calcd for $C_{20}H_{12}Br_2O_4^+$ 473.9103; found 473.9093.

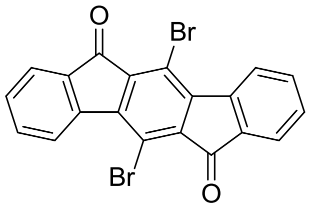

5,11-Dibromoindeno[1,2-*b*]fluorene-6,12-dione (11). Terephthalic acid **15** (1.97 g, 4.13 mmol) was slowly added to stirred conc. H_2SO_4 (130 mL) which resulted in a rapid color changed to opaque dark brown. After stirring at rt for 16 h, the reaction mixture was poured slowly onto ice (ca. 400 g). The precipitate was collected and washed sequentially with H_2O, satd $NaHCO_3$ solution, H_2O, and then acetone. The solid was dried overnight at 70 °C to yield **11** (1.36 g, 3.08 mmol, 75%) as a bright orange solid. ^{1}H NMR (600 MHz, $CDCl_3$) δ 8.67 (d, J = 7.7 Hz, 2H), 7.77 (d, J =7.2 Hz, 2H), 7.62 (dt, J = 7.6, 1.3 Hz, 2H), 7.44 (t, J = 7.5 Hz, 2H); ^{13}C NMR (151 MHz, $CDCl_3$): δ 189.92, 147.02, 141.38, 137.30, 135.42, 134.22, 130.49, 124.95, 124.78, 115.14; HRMS (TOF MS ESI+) $[M]^+$ calcd for $C_{20}H_8Br_2O_2^+$ 437.8891; found 437.8871.

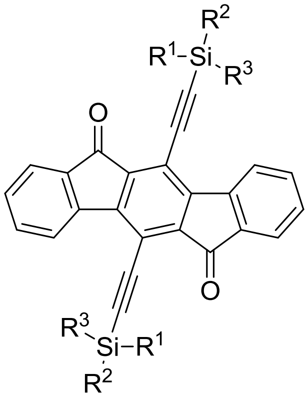

Cross-coupling with 5,11-diiodoindeno[1,2-*b*]fluorene-6,12-dione. A mixture of dione **9** (0.100 g, 0.187 mmol), $Pd(PPh_3)_4$ (0.025 g, 0.022 mmol) and CuI (0.002 g, 0.011 mmol) in THF (30 mL) and $i Pr_2NH$ (30 mL) was degassed with Ar for 45 min. The appropriate ethynylsilane (0.748 mmol) was then added via syringe if it was a liquid. Alternatively, solid triphenylsilylacetylene was dissolved in THF (6 mL), degassed by Ar bubbling for 30 min and then transferred via cannula under inert atmosphere. The reaction mixture was then heated to 55-60 °C and monitored via NMR until complete, typically 24-48 h. After removal of the solvent, the residue was purified as indicated to give product **8** as an orange solid. The spectral data for **8a-b,d-f,i** are given in reference 7.

Cross-coupling with 5,11-Dibromoindeno[1,2-*b*]fluorene-6,12-dione. A pressure reaction vessel charged with dione **11** (0.73 g, 1.6 mmol), CuI (0.02 g, 0.08 mmol), $Pd(PPh_3)_2Cl_2$ (0.120 g, 0.17 mmol) in Et_3N (15 mL) and THF (10 mL) was degassed with by evacuating and backfilling the reaction vessel with Ar (3x). The appropriate ethynylsilane (8.3 mmol) was added and the sealed flask was stirred at 100 °C for 36 h. After cooling to rt, the solvent was removed in vacuo and the residue was purified by column chromatography on silica (see below for eluent conditions) to give product **8** as an orange solid. Analytical samples were obtained using a recycling HPLC with polystyrene beads eluting with $CHCl_3$.

8b ($SiEt_3$). 4:1 hexanes/CH_2Cl_2; 27% yield. NMR data matched those in reference 7.

8c ($SiPr_3$). 4:1 hexanes/CH_2Cl_2; 72% yield. ^{1}H NMR (600 MHz, $CDCl_3$) δ 8.59 (d, *J* = 7.6 Hz, 2H), 7.72 (d, *J* = 7.3 Hz, 2H), 7.51 (td, *J* = 7.5, 1.3 Hz, 2H), 7.39 (t, *J* = 7.4 Hz, 2H), 1.61

(m, 12H), 1.07 (t, J = 7.3 Hz, 18H), 0.86 (m, 12H); ^{13}C NMR (151 MHz, $CDCl_3$): δ 190.42, 146.63, 142.34, 138.60, 134.71, 134.11, 129.91, 124.31, 123.66, 114.24, 108.45, 99.98, 18.58, 17.80, 16.09; UV/Vis ($CHCl_3$) λ_{max} 312, 332, 524 nm ; HRMS (TOF MS ESI+) for $C_{42}H_{51}O_2Si_2^+$ [M^++H]: calcd 643.3428; found 643.3397.

8d ($Si(iPr)_3$). 9:1 hexanes/CH_2Cl_2; 48% yield. NMR data matched those listed above.

8g ($SiMe_2(CH_2)_2CF_3$). 4:1 hexanes/CH_2Cl_2 increasing polarity to 7:3 hexanes/CH_2Cl_2; 30% yield. ^{1}H NMR (600 MHz, $CDCl_3$) δ 8.45 (d, J = 7.6 Hz, 2H), 7.72 (d, J = 7.7 Hz, 2H), 7.54 (dt, J = 7.6, 1.2 Hz, 2H), 7.40 (dt, J = 7.5, 0.8 Hz , 2H), 2.39 (m, 4H), 1.08 (m, 4H), 0.45 (s, 12H); ^{13}C NMR (151 MHz, $CDCl_3$): δ 190.28, 146.65, 142.08, 138.75, 135.06, 134.06, 130.25, 124.51, 123.39, 113.87, 106.74, 100.35, 29.0 (q, J = 30.1 Hz), 8.16 (q, J = 2.2 Hz), –2.06, –3.63; UV/Vis ($CHCl_3$) λ_{max} 308, 330, 516 nm; HRMS (TOF MS ESI+) for $C_{34}H_{29}O_2F_6Si_2^+$ [M^++H]: calcd 639.1610; found 639.1606.

8h ($SiMe_2iBu$). 4:1 hexanes/CH_2Cl_2; 35% yield. ^{1}H NMR (600 MHz, $CDCl_3$) δ 8.54 (d, J = 7.7 Hz, 2H), 7.71 (d, J = 7.2 Hz, 2H), 7.53 (dt, J = 7.6, 1.3 HZ, 2H), 7.37 (dt, J = 7.4, 1.0 Hz, 2H), 2.03 (n, J = 6.7 Hz 2H), 1.08 (d, J = 6.6 Hz, 12H), 0.88 (d, J = 6.6 Hz, 4H), 0.43 (s, 12H); ^{13}C NMR (151 MHz, $CDCl_3$): δ 190.51, 146.61, 142.29, 138.57, 134.86, 134.08, 129.96, 124.28, 123.62, 114.23, 109.54, 99.43, 26.64, 26.43, 25.36, 0.76; UV/Vis ($CHCl_3$) λ_{max} 311, 331, 523 nm; HRMS (TOF MS ESI+) for $C_{36}H_{39}O_2Si_2^+$ [M^++H]: calcd 559.2489; found 559.2502.

8j ($SiMe_2Ph$). 4:1 hexanes/CH_2Cl_2; 24% yield. ^{1}H NMR (600 MHz, $CDCl_3$) δ 8.37 (m, 2H), 7.82 (m, 4H), 7.70 (m, 2H), 7.45 (m, 6H), 7.34 (m, 4H), 0.68 (s, 12H); ^{13}C NMR (151 MHz, $CDCl_3$): δ 190.50, 146.82, 138.64, 136.06, 135.03, 134.20, 134.02, 130.01, 129.98, 128.34, 124.30, 123.73, 114.09, 107.53, 100.34, 10.7; UV/Vis ($CHCl_3$) λ_{max} 310, 331, 521 nm; HRMS (TOF MS ESI+) $[M]^+$ for $C_{40}H_{31}O_2Si_2^+$: calcd 599.1863; found 599.1870.

Electronic Absorption Spectra

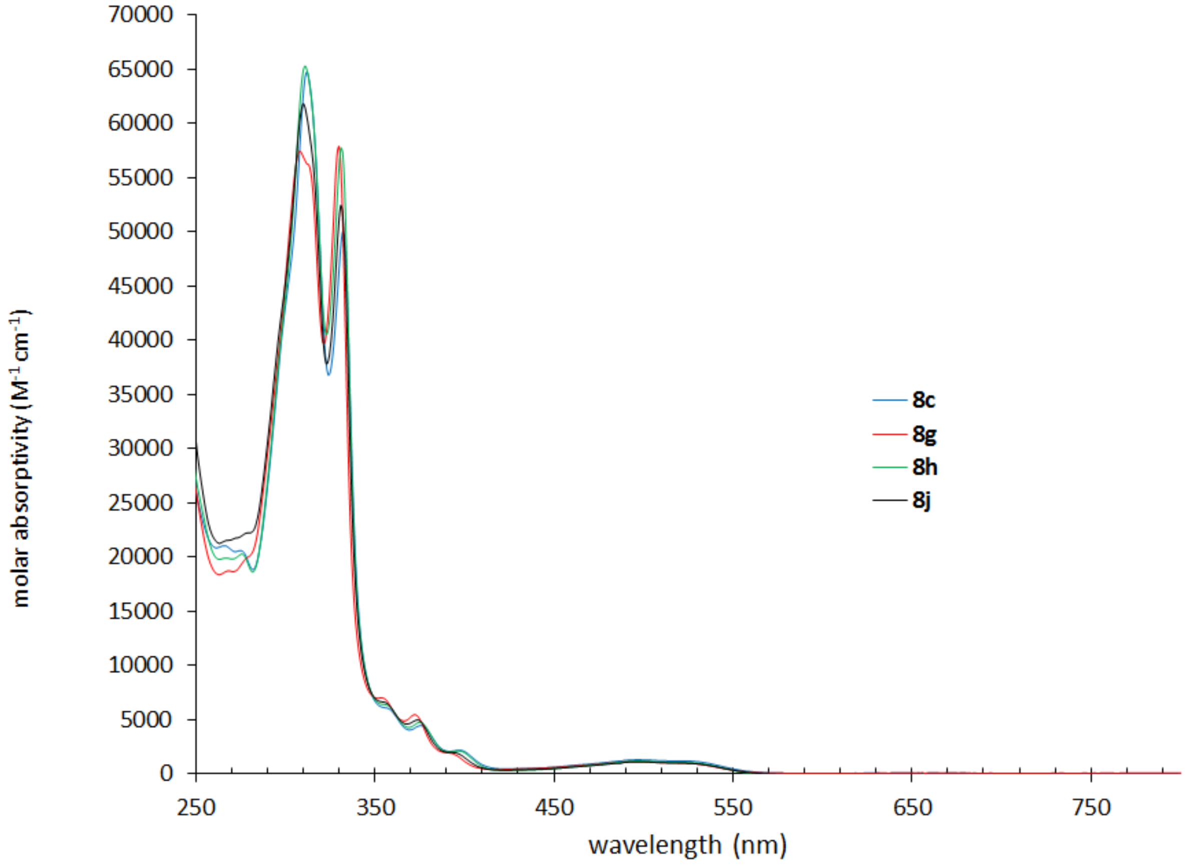

Figure S1: Electronic absorption spectra of diones **8c**, **8g**, **8h** and **8j**.

Electrochemical details

All electrochemical experiments were conducted in a traditional 3-electrode geometry using a Solartron 1287 potentiostat. Electrolyte solutions (0.1 M) were prepared from HPLC-grade CH_2Cl_2 and anhydrous Bu_4NBF_4, and the solutions were freeze-pump-thaw degassed (3x) prior to analysis. Cyclic voltammetry was conducted under a nitrogen atmosphere. The working electrode was a glassy carbon electrode (3-mm diameter), with a Pt-coil counter electrode and Ag wire pseudo reference. The ferrocene/ferrocenium (Fc/Fc^+) couple was used as an internal standard following each experiment. Potential values were re-referenced to SCE using a value of 0.46 (V vs. SCE) for the Fc/Fc^+ couple in CH_2Cl_2. When necessary, potentials were re-referenced to NHE using SCE = –0.24 (V vs. NHE). LUMO energy levels were approximated using SCE = –4.68 eV vs. vacuum.[4] Cyclic voltammetry experiments were conducted at sweep rates of 50 (reported), 75, 100 and 125 mV s^{-1}. All scan rates show quasi-reversible kinetics with no alteration of peak splitting with scan rate. $E_{1,2}$ values were calculated assuming $E_{1/2} \approx E^{o'}=(E_{anodic} + E_{cathodic})/2$ based on these observations. The $E_{a,c}$ peak splitting of the Fc/Fc^+ couple was similar to that of the analyte (~100 mV). The anodic peak current increases linearly with the square root of the scan rate in the range 50 to 125 mV s^{-1}, indicating a diffusion-controlled process. Analyte concentrations were ca. 5-7 mM.

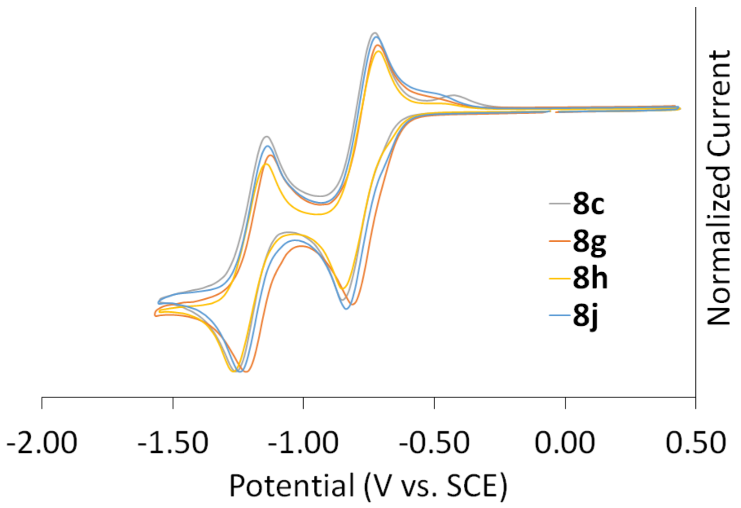

Figure S2: Cyclic voltammogtrams of **8c**, **8g**, **8h** and **8j**.

X-ray crystallography

Diffraction intensities for **8b**, **8e, 8i; 8c, 8h** and **8g, 8j** were collected at 173(2); 193(2) and 100(2) K, respectively, on a Bruker Apex2 CCD diffractometer using MoKα radiation λ = 0.71073 Å. Space groups were determined based on systematic absences (**8b**, **8e, 8i; 8g, 8h**) and intensity statistics (**8c** and **8j**). Absorption corrections were applied by SADABS.[5] Structures were solved by direct methods and Fourier techniques and refined on F^2 using full matrix least-squares procedures. All non-H atoms were refined with anisotropic thermal parameters. All H atoms in **8b**, **8e** and **8i** were found from the residual density map and refined with isotropic thermal parameters. H atoms in **8c, 8g, 8h** and **8j** were refined in calculated positions in a rigid group model. All calculations were performed by the Bruker SHELXTL (v. 6.10) package.[6] These data can be obtained free of charge from The Cambridge Crystallographic Data Centre via www.ccdc.cam.ac.uk/data_request/cif. The x-ray data for **8a** (CCDC 841548), **8d** (CCDC 787155) and **8f** (CCDC 841549) were reported in the original communication.[7]

Crystallographic data for **8b**: $C_{36}H_{38}O_2Si_2$, M = 558.84, 0.35 x 0.11 x 0.12 mm, T = 173(2) K, Monoclinic, space group $P2_1/c$, a = 17.3760(18) Å, b = 6.4393(7) Å, c = 15.6311(16) Å, β = 116.306(2)°, V = 1567.8(3) Å^3, Z = 2, D_c = 1.184 Mg/m^3, μ = 0.143 mm^{-1}, F(000) = 596, $2\theta_{max}$ = 54.00°, 12383 reflections, 3420 independent reflections [R_{int} = 0.0211], R1 = 0.0401, wR2 = 0.1106 and GOF = 1.034 for 3420 reflections (257 parameters) with I>2σ(I), R1 = 0.0460, wR2 = 0.1169 and GOF = 1.034 for all reflections, max/min residual electron density +0.398/–0.214 eÅ^{-3}. CCDC 1011434.

Crystallographic data for **8c**: $C_{42}H_{50}O_2Si_2$, M = 643.00, 0.46 x 0.24 x 0.03 mm, T = 193(2) K, Triclinic, space group P-1, a = 8.7419(11) Å, b = 12.1839(16) Å, c = 18.359(2) Å, α = 106.489(2)°, β = 92.556(2)°, γ = 94.239(2)°, V = 1865.5(4) Å^3, Z = 2, D_c = 1.145 Mg/m^3, μ = 0.129 mm^{-1}, F(000) = 692, $2\theta_{max}$ = 50.00°, 17919 reflections, 6560 independent reflections [R_{int} = 0.0345], R1 = 0.0682, wR2 = 0.1686 and GOF = 1.055 for 6560 reflections (411 parameters)

with I>2σ(I), R1 = 0.1071, wR2 = 0.2015 and GOF = 1.058 for all reflections, max/min residual electron density +0.697/–0.285 eÅ^{-3}. CCDC 1011438.

Crystallographic data for **8e**: $C_{48}H_{62}O_2Si_2$, M = 727.16, 0.45 x 0.07 x 0.05 mm, T = 173(2) K, Monoclinic, space group $P2_1/n$, a = 6.1344(6) Å, b = 17.3347(17) Å, c = 20.190(2) Å, β = 90.915(2)°, V = 2146.7(4) Å^3, Z = 2, D_c = 1.125 Mg/m^3, μ = 0.119 mm^{-1}, F(000) = 788, $2\theta_{max}$ = 50.0°, 13428 reflections, 3784 independent reflections [R_{int} = 0.0513], R1 = 0.0462, wR2 = 0.1035 and GOF = 1.011 for 37845 reflections (359 parameters) with I>2σ(I), R1 = 0.0723, wR2 = 0.1185 and GOF = 1.011 for all reflections, max/min residual electron density +0.315/–0.281 eÅ^{-3}. CCDC 1011435.

Crystallographic data for **8g**: $C_{34}H_{28}F_6O_3Si_2$, M = 638.74, 0.14 x 0.12 x 0.09 mm, T = 100(2) K, Orthorhombic, space group $Pca2_1$, a = 15.7713(9) Å, b = 6.0958(4) Å, c = 32.7766(19) Å, V = 3151.1(3) Å^3, Z = 4, D_c = 1.346 Mg/m^3, μ = 0.178 mm^{-1}, F(000) = 1320, $2\theta_{max}$ = 56.00°, 15175 reflections, 7303 independent reflections [R_{int} = 0.0253], R1 = 0.0452, wR2 = 0.1040 and GOF = 1.059 for 7303 reflections (397 parameters) with I>2σ(I), R1 = 0.0558, wR2 = 0.1088 and GOF = 1.059 for all reflections, the Flack = 0.03(11), max/min residual electron density +0.420/–0.321 eÅ^{-3}. CCDC 1011439.

Crystallographic data for **8h**: $C_{36}H_{38}O_2Si_2$, M = 558.84, 0.35 x 0.07 x 0.05 mm, T = 193(2) K, Monoclinic, space group $P2_1/c$, a = 16.489(6) Å, b = 5.976(2) Å, c = 16.259(5) Å, V = 1579.3(9) Å^3, Z = 2, D_c = 1.175 Mg/m^3, μ = 0.142 mm^{-1}, F(000) = 596, $2\theta_{max}$ = 50.00°, 13905 reflections, 2775 independent reflections [R_{int} = 0.0697], R1 = 0.0568, wR2 = 0.1409 and GOF = 1.056 for 2775 reflections (181 parameters) with I>2σ(I), R1 = 0.0919, wR2 = 0.1690 and GOF = 1.056 for all reflections, max/min residual electron density +0.307/–0.315 eÅ^{-3}. CCDC 1011437.

Crystallographic data for **8i**: $C_{36}H_{38}O_2Si_2$, M = 558.84, 0.42 x 0.20 x 0.03 mm, T = 173(2) K, Monoclinic, space group $P2_1/c$, a = 16.742(4) Å, b = 6.3559(14) Å, c = 15.144(3) Å, β = 100.795(4)°, V = 1582.9(6) Å^3, Z = 2, D_c = 1.173 Mg/m^3, μ = 0.142 mm^{-1}, F(000) = 596, $2\theta_{max}$ =

54.00°, 14104 reflections, 3449 independent reflections [R_{int} = 0.0386], R1 = 0.0460, wR2 = 0.1169 and GOF = 1.097 for 3449 reflections (257 parameters) with I>2σ(I), R1 = 0.0632, wR2 = 0.1371 and GOF = 1.097 for all reflections, max/min residual electron density +0.420/–0.278 e$Å^{-3}$. CCDC 1011436.

Crystallographic data for **8j**: $C_{40}H_{30}O_2Si_2$, M = 598.82, 0.32 x 0.12 x 0.04 mm, T = 100(2) K, Triclinic, space group *P*-1, a = 6.2923(12) Å, b = 15.615(3) Å, c = 16.303(3) Å, α = 103.724(2)°, β = 90.601(3)°, γ = 93.428(2)°, V = 1552.9(5) $Å^3$, Z = 2, D_c = 1.281 Mg/m^3, μ = 0.150 mm^{-1}, F(000) = 628, $2\theta_{max}$ = 50.00°, 18419 reflections, 5434 independent reflections [R_{int} = 0.0503], R1 = 0.0399, wR2 = 0.0891 and GOF = 1.014 for 5434 reflections (397 parameters) with I>2σ(I), R1 = 0.0632, wR2 = 0.0993 and GOF = 1.014 for all reflections, max/min residual electron density +0.281/–0.295 e$Å^{-3}$. CCDC 1011440.

Computational details

Calculations were performed in Gaussian 09[8] and results were visualized in Gaussview 5.[9] All minimized geometries were verified as minima using analytically obtained frequencies ensuring that all frequencies were positive. Geometries were minimized using B3LYP[10–12] functional with the 6-31G(d) basis set. Time dependent single point energies were then carried out using B3LYP functional and 6-311+G(d,p) basis set. Energies are given in Hartrees.

Fluorenone

E(RB3LYP) = -575.437041862

Zero-point correction = 0.169993 (Hartree/Particle)

Sum of electronic and zero-point Energies = -575.267049

Atomic Number	X	Y	Z
6	3.031563	-1.393089	-0.000144
6	3.468261	-0.065168	-0.000144

6	2.539066	0.983719	-0.000091
6	1.189461	0.665408	-0.000039
6	0.742036	-0.671224	-0.000038
6	1.664659	-1.710876	-0.00009
1	3.763676	-2.196219	-0.000186
1	4.532617	0.151689	-0.000185
1	2.854581	2.023303	-0.000091
1	1.345666	-2.74975	-0.00009
6	0	1.577717	0.000021
6	-1.189461	0.665408	0.000058
6	-2.539066	0.983719	0.000116
6	-3.468261	-0.065169	0.000142
6	-3.031563	-1.393089	0.000105
6	-1.664659	-1.710876	0.000046
6	-0.742036	-0.671224	0.000023
1	-2.854581	2.023303	0.000142
1	-4.532618	0.151689	0.00019
1	-3.763676	-2.196219	0.000123
1	-1.345666	-2.74975	0.00002
8	0	2.796303	0.000036

Benzophenone

E(RB3LYP) = -576.632267849

Zero-point correction = 0.192039 (Hartree/Particle)

Sum of electronic and zero-point Energies = -576.440229

Atomic Number	X	Y	Z
6	0.000014	1.080491	-0.000004
8	0.000002	2.306866	-0.000052
6	-1.303858	0.337286	0.024491
6	-1.454265	-0.925657	0.617795
6	-2.434074	0.983323	-0.502109
6	-2.708843	-1.534408	0.67056
1	-0.596683	-1.421123	1.061536
6	-3.680729	0.36645	-0.466685
1	-2.310177	1.971543	-0.933156
6	-3.820701	-0.894964	0.120505
1	-2.817285	-2.506434	1.144026
1	-4.546443	0.868969	-0.88957
1	-4.795612	-1.374137	0.154485
6	1.303909	0.337347	-0.02443

6	1.454234	-0.925562	-0.617824
6	2.434141	0.983322	0.50218
6	2.708769	-1.534394	-0.670609
1	0.596592	-1.420922	-1.061583
6	3.680761	0.366374	0.466719
1	2.310287	1.971521	0.933288
6	3.820658	-0.895025	-0.120521
1	2.817163	-2.506407	-1.144111
1	4.54651	0.868823	0.889617
1	4.795543	-1.374249	-0.154517

Dione 7a

E(RB3LYP) = -1888.30699292

Zero-point correction = 0.462882 (Hartree/Particle)

Sum of electronic and zero-point Energies = -1887.844111

Atomic Number	X	Y	Z
6	1.450667	0.032662	0.001002
6	0.664894	1.202577	0.000617
6	0.742202	-1.189641	0.000258
6	-0.664898	-1.202551	-0.000627
6	-1.450671	-0.032634	-0.001011
6	-0.742206	1.189668	-0.00027
6	1.130061	2.636739	0.00074
6	-1.234289	2.590735	-0.000334
6	-1.130065	-2.636713	-0.000742
8	-2.274835	-3.051807	-0.001258
8	2.274831	3.051832	0.001262
6	0.119251	-3.451679	-0.000121
6	1.234286	-2.590708	0.000319
6	-0.119255	3.451706	0.000116
6	2.518612	-3.128163	0.000464
1	3.392674	-2.488402	0.000495
6	2.657095	-4.524854	0.000355
1	3.655101	-4.955142	0.000374
6	-2.518616	3.12819	-0.000494
1	-3.392677	2.488429	-0.00054
6	-2.657099	4.52488	-0.000383
1	-3.655105	4.955169	-0.000412
6	-1.545073	5.370916	-0.000113
1	-1.685127	6.44813	-0.000034

6	1.545069	-5.370889	0.000099
1	1.685123	-6.448103	0.000021
6	0.252422	-4.83124	-0.000191
1	-0.630932	-5.46355	-0.000521
6	-0.252426	4.831267	0.000188
1	0.630928	5.463577	0.000526
6	-2.871701	-0.103024	-0.001918
6	2.871697	0.103048	0.001915
6	4.089686	0.189218	0.002527
6	-4.089689	-0.189205	-0.002525
14	-5.901489	-0.540714	0.000351
14	5.901495	0.540674	-0.000334
6	-6.260267	-1.701436	1.445344
1	-5.665448	-2.61764	1.363489
1	-6.018595	-1.229914	2.40459
1	-7.320318	-1.983516	1.46681
6	-6.338468	-1.36077	-1.64365
1	-7.405562	-1.613118	-1.681021
1	-6.119842	-0.701038	-2.490897
1	-5.767091	-2.285088	-1.781974
6	-6.846058	1.084896	0.202073
1	-6.576979	1.590873	1.136396
1	-6.641157	1.774037	-0.625407
1	-7.927729	0.903083	0.222316
6	6.26032	1.701408	-1.445306
1	6.018615	1.22992	-2.40456
1	5.665553	2.617644	-1.363426
1	7.320386	1.983431	-1.466776
6	6.846009	-1.084966	-0.202075
1	7.927688	-0.903194	-0.222291
1	6.641064	-1.774118	0.625384
1	6.57693	-1.590909	-1.136415
6	6.33849	1.360694	1.64368
1	5.767141	2.285029	1.78201
1	6.119836	0.70096	2.490918
1	7.405591	1.613008	1.681062

References

1. Zhou, Q.; Carroll, P. J.; Swager, T. M. *J. Org. Chem.* **1994**, *59*, 1294–1301.
2. Lehnherr, D.; Murray, A. H.; McDonald, R.; Tykwinski, R. R. *Angew. Chem. Int. Ed.* **2010**, *9999*, NA.
3. Anthony, J. E.; Eaton, D. L.; Parkin, S. R. *Org. Lett.* **2002**, *4*, 15–18.
4. Reiss, H.; Heller, A. *J. Phys. Chem.* **1985**, *89*, 4207–4213.
5. Sheldrick, G. M. *Bruker/Siemens Area Detector Absorption Correction Program*, Bruker AXS, Madison, WI, 1998.
6. SHELXTL-6.10 "Program for Structure Solution, Refinement and Presentation" BRUKER AXS Inc., 5465 East Cheryl Parkway, Madison, WI 53711-5373 USA.
7. Rose, B. D.; Chase, D. T.; Weber, C. D.; Zakharov, L. N.; Lonergan, M. C.; Haley, M. M. *Org. Lett.* **2011**, *13*, 2106–2109.
8. Frisch, M. J.; Trucks, G. W.; Schlegel, H. B.; Scuseria, G. E.; Robb, M. A.; Cheeseman, J. R.; Scalmani, G.; Barone, V.; Mennucci, B.; Petersson, G. A.; Nakatsuji, H.; Caricato, M.; Li, X.; Hratchian, H. P.; Izmaylov, A. F.; Bloino, J.; Zheng, G.; Sonnenberg, J. L.; Hada, M.; Ehara, M.; Toyota, K.; Fukuda, R.; Hasegawa, J.; Ishida, M.; Nakajima, T.; Honda, Y.; Kitao, O.; Nakai, H.; Vreven, T.; Montgomery, Jr., J. A.; Peralta, J. E.; Ogliaro, F.; Bearpark, M.; Heyd, J. J.; Brothers, E.; Kudin, K. N.; Staroverov, V. N.; Kobayashi, R.; Normand, J.; Raghavachari, K.; Rendell, A.; Burant, J. C.; Iyengar, S. S.; Tomasi, J.; Cossi, M.; Rega, N.; Millam, N. J.; Klene, M.; Knox, J. E.; Cross, J. B.; Bakken, V.; Adamo, C.; Jaramillo, J.; Gomperts, R.; Stratmann, R. E.; Yazyev, O.; Austin, A. J.; Cammi, R.; Pomelli, C.; Ochterski, J. W.; Martin, R. L.; Morokuma, K.; Zakrzewski, V. G.; Voth, G. A.; Salvador, P.; Dannenberg, J. J.; Dapprich, S.; Daniels, A. D.; Farkas, Ö.; Foresman, J. B.; Ortiz, J. V.; Cioslowski, J.; Fox, D. J. *Gaussian 09*; 2010.
9. Dennington, R.; Keith, T.; Millam, J. *GaussView*; Semichem Inc.: Shawnee Mission KS, 2009.
10. Becke, A. D. *J. Chem. Phys.* **1993**, *98*, 5648–5652.
11. Lee, C.; Yang, W.; Parr, R. G. *Phys Rev B Condens Matter* **1988**, *37*, 785–789.
12. Stephens, P. J.; Devlin, F. J.; Chabalowski, C. F.; Frisch, M. J. *J. Phys. Chem.* **1994**, *98*, 11623–11627.

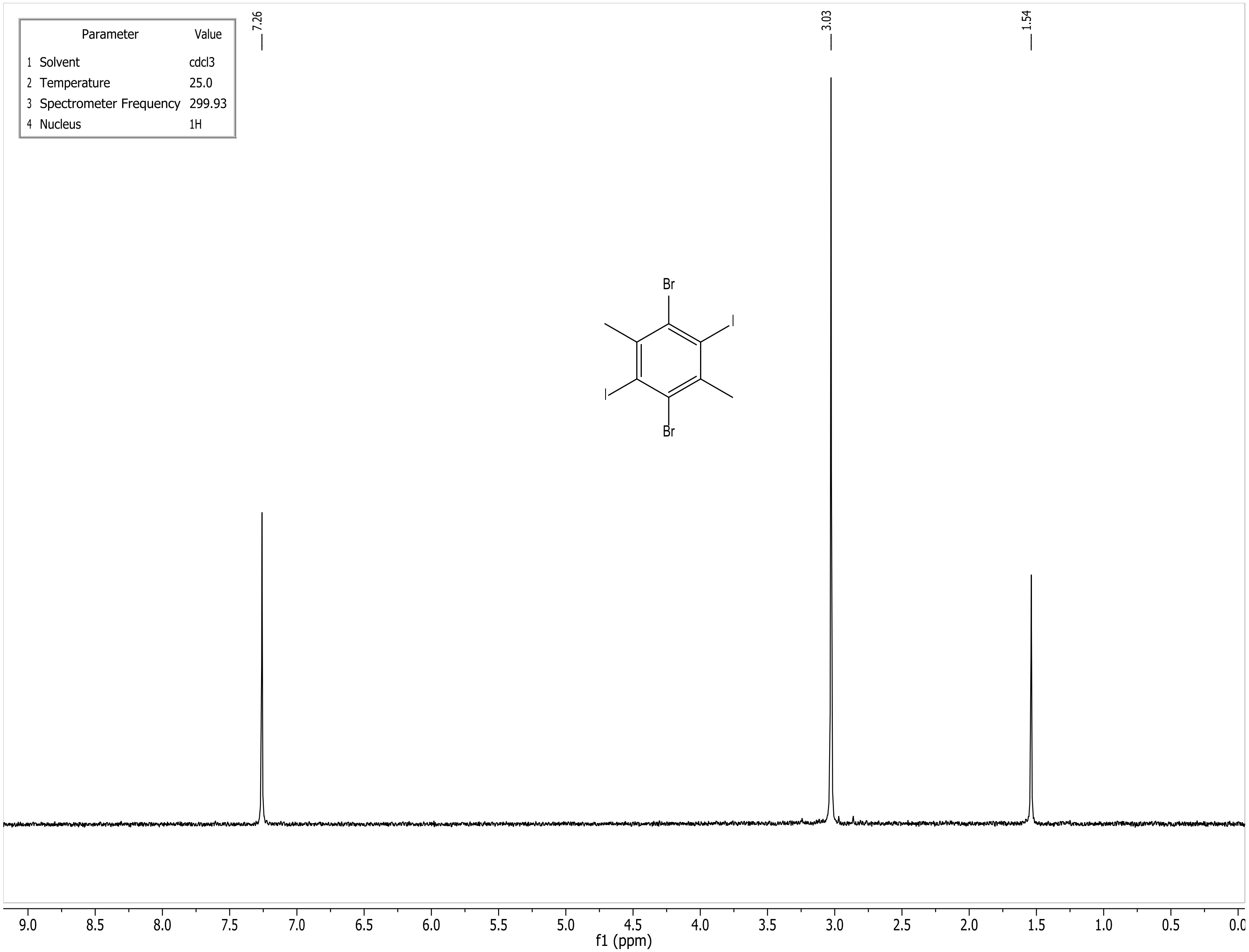

Parameter Value
1 Solvent cdcl3
2 Temperature 25.0
3 Spectrometer Frequency 299.93
4 Nucleus 1H
7.26
3.03
1.54
Br
I
I
Br
9.0 8.5 8.0 7.5 7.0 6.5 6.0 5.5 5.0 4.5 4.0 3.5 3.0 2.5 2.0 1.5 1.0 0.5 0.0
f1 (ppm)

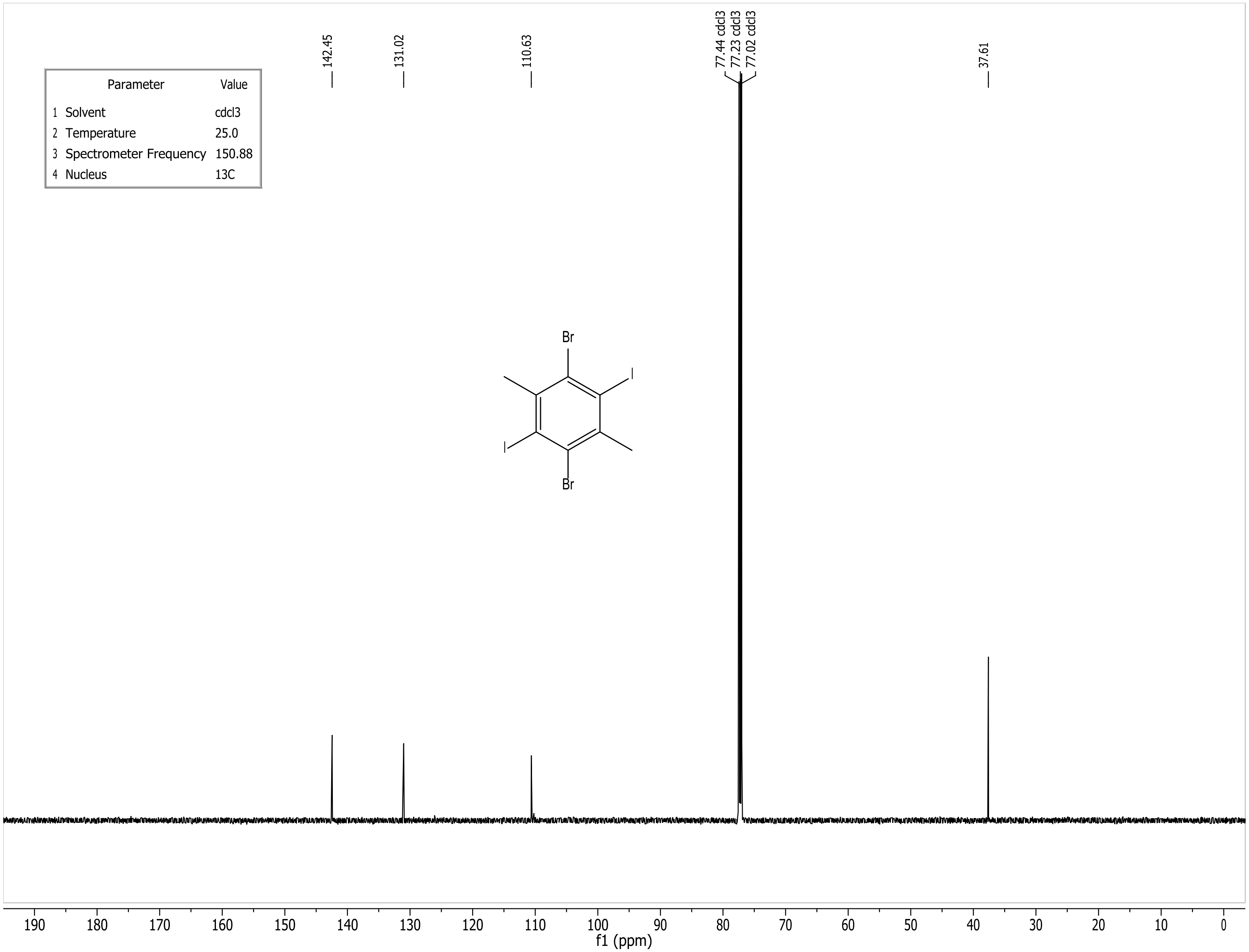

Parameter Value
1 Solvent cdcl3
2 Temperature 25.0
3 Spectrometer Frequency 150.88
4 Nucleus 13C
142.45
131.02
110.63
77.44 cdcl3
77.23 cdcl3
77.02 cdcl3
37.61
Br
I
I
Br
190 180 170 160 150 140 130 120 110 100 90 80 70 60 50 40 30 20 10 0
f1 (ppm)

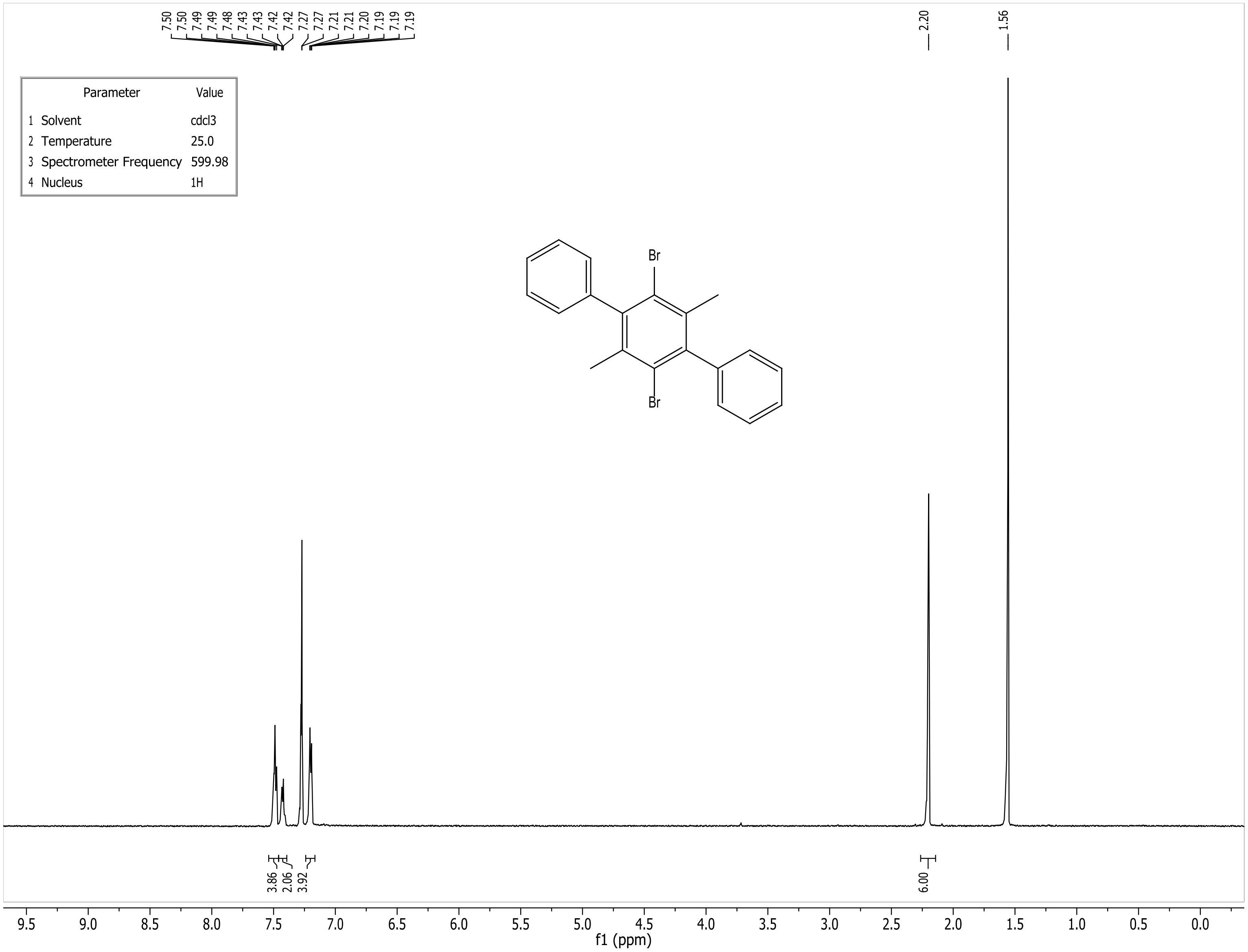
7.50
7.50
7.49
7.49
7.48
7.43
7.43
7.42
7.42
7.27
7.27
7.21
7.21
7.20
7.19
7.19
7.19
2.20
1.56
Parameter
Value
1 Solvent
cdcl3
2 Temperature
25.0
3 Spectrometer Frequency
599.98
4 Nucleus
1H
Br
Br
3.86
2.06
3.92
6.00
9.5
9.0
8.5
8.0
7.5
7.0
6.5
6.0
5.5
5.0
4.5
4.0
3.5
3.0
2.5
2.0
1.5
1.0
0.5
0.0
f1 (ppm)

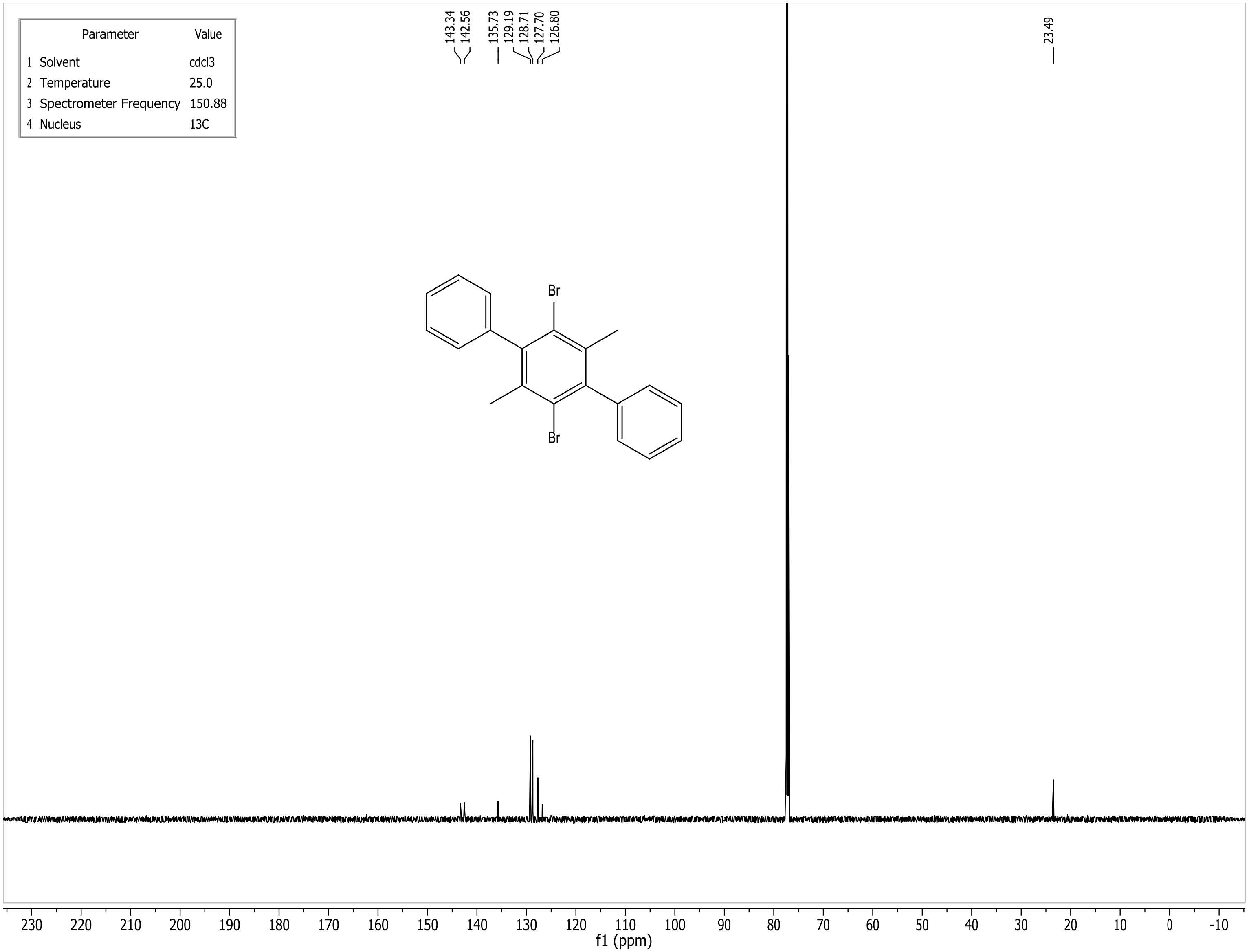
Parameter Value
1 Solvent cdcl3
2 Temperature 25.0
3 Spectrometer Frequency 150.88
4 Nucleus 13C
143.34
142.56
135.73
129.19
128.71
127.70
126.80
23.49
Br
Br
230 220 210 200 190 180 170 160 150 140 130 120 110 100 90 80 70 60 50 40 30 20 10 0 -10
f1 (ppm)

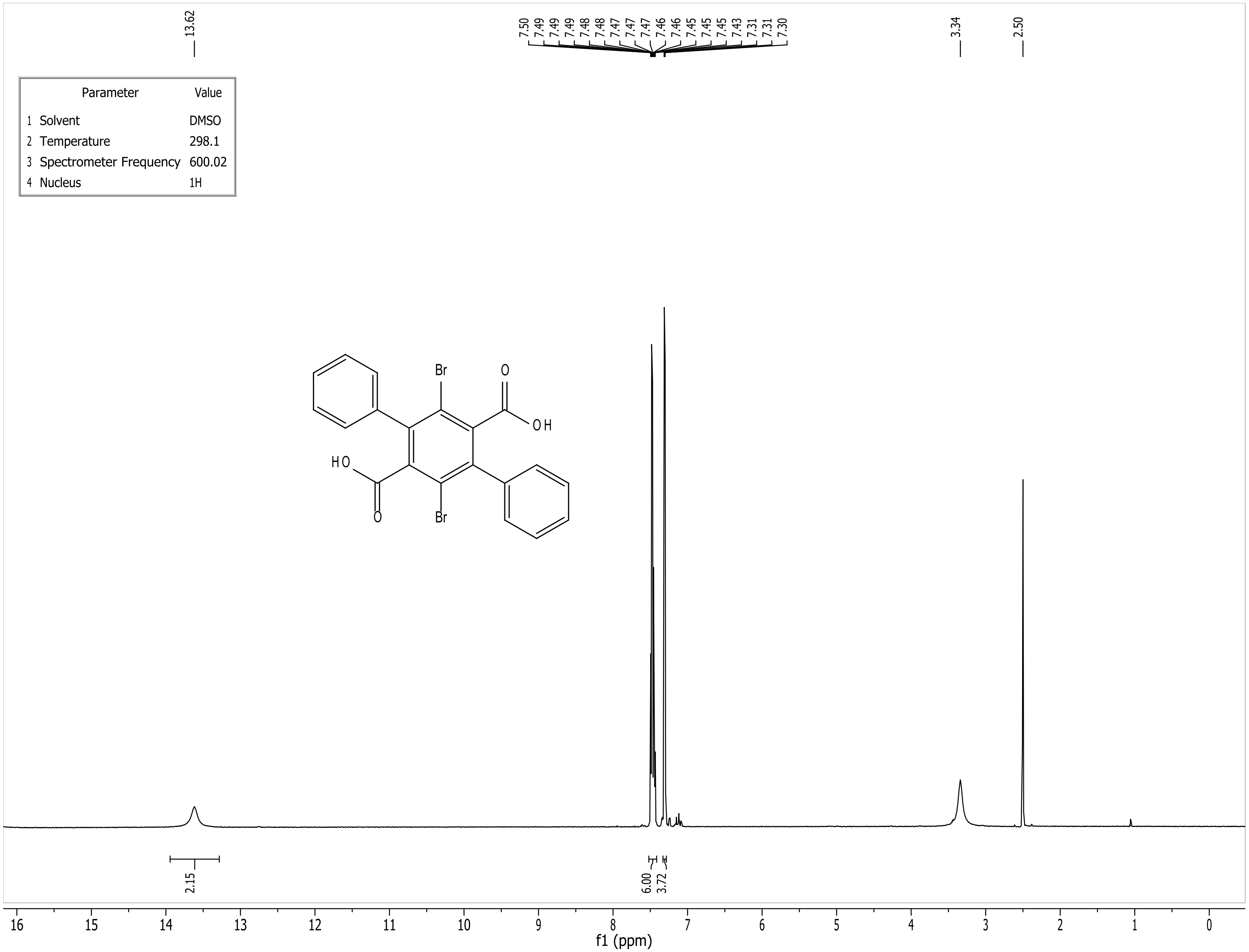
13.62
7.50 7.49 7.49 7.49 7.48 7.48 7.47 7.47 7.47 7.46 7.46 7.45 7.45 7.45 7.43 7.31 7.31 7.30
3.34
2.50
Parameter Value
1 Solvent DMSO
2 Temperature 298.1
3 Spectrometer Frequency 600.02
4 Nucleus 1H
Br
O
OH
HO
O
Br
2.15
6.00
3.72
16 15 14 13 12 11 10 9 8 7 6 5 4 3 2 1 0
f1 (ppm)

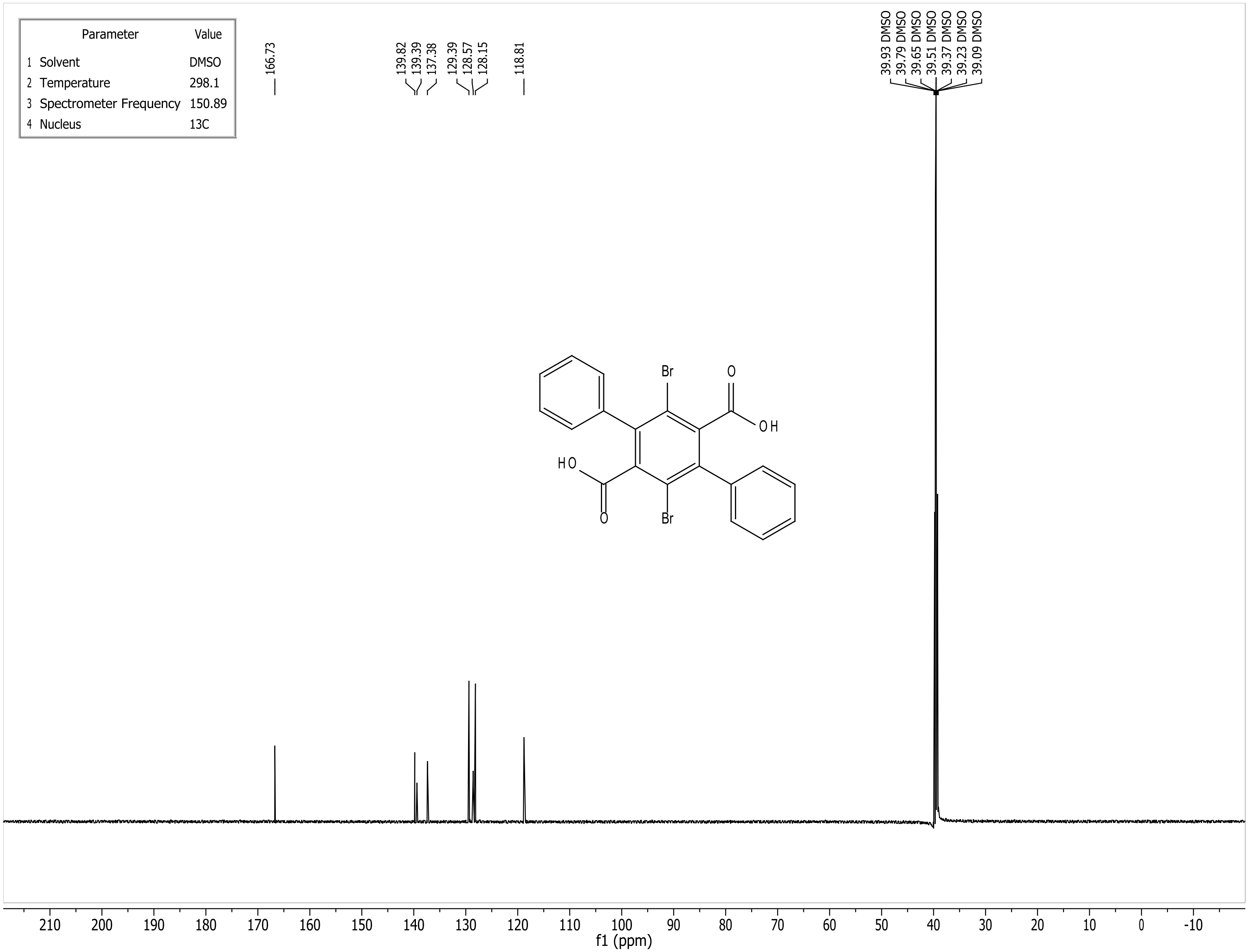
Parameter Value
1 Solvent DMSO
2 Temperature 298.1
3 Spectrometer Frequency 150.89
4 Nucleus 13C
166.73
139.82
139.39
137.38
129.39
128.57
128.15
118.81
39.93 DMSO
39.79 DMSO
39.65 DMSO
39.51 DMSO
39.37 DMSO
39.23 DMSO
39.09 DMSO
Br
O
OH
HO
O
Br
210 200 190 180 170 160 150 140 130 120 110 100 90 80 70 60 50 40 30 20 10 0 -10
f1 (ppm)

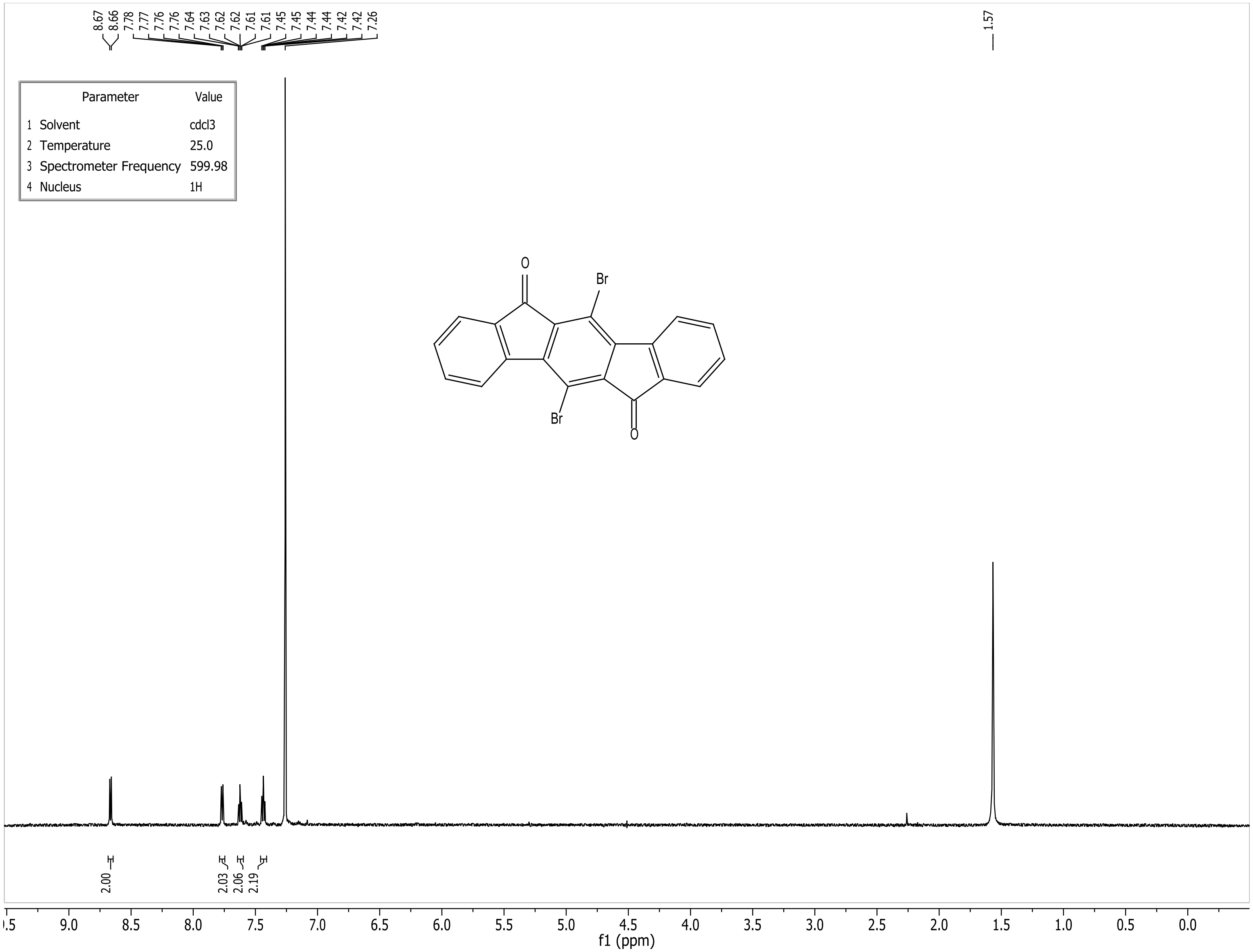
8.67
8.66
7.78
7.77
7.76
7.76
7.64
7.63
7.62
7.62
7.61
7.61
7.45
7.45
7.44
7.44
7.42
7.42
7.26
1.57
Parameter Value
1 Solvent cdcl3
2 Temperature 25.0
3 Spectrometer Frequency 599.98
4 Nucleus 1H
O
Br
Br
O
2.00
2.03
2.06
2.19
9.0
8.5
8.0
7.5
7.0
6.5
6.0
5.5
5.0
4.5
4.0
3.5
3.0
2.5
2.0
1.5
1.0
0.5
0.0
f1 (ppm)

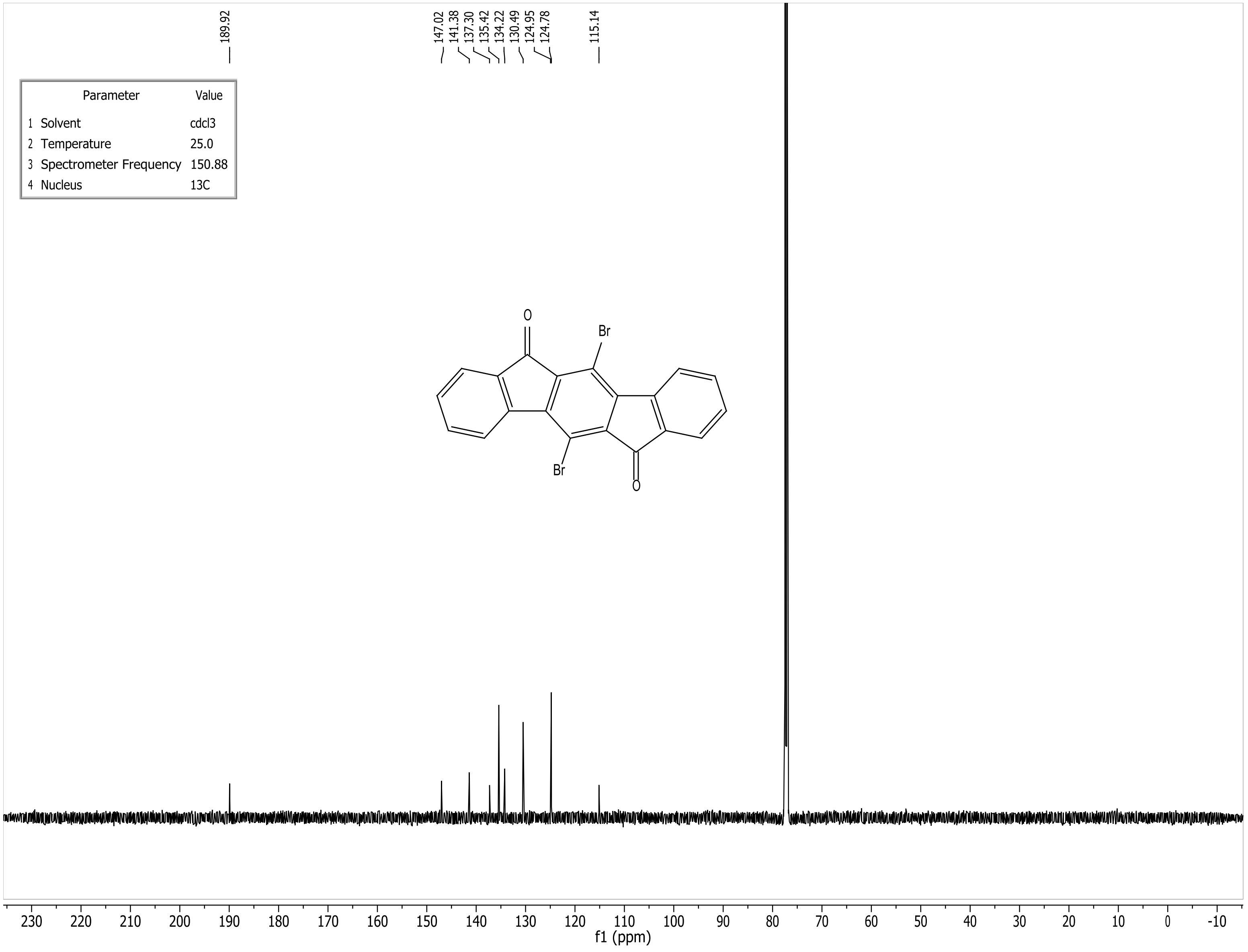
189.92
147.02
141.38
137.30
135.42
134.22
130.49
124.95
124.78
115.14
Parameter
Value
1 Solvent
cdcl3
2 Temperature
25.0
3 Spectrometer Frequency
150.88
4 Nucleus
13C
O
Br
Br
O
230 220 210 200 190 180 170 160 150 140 130 120 110 100 90 80 70 60 50 40 30 20 10 0 -10
f1 (ppm)

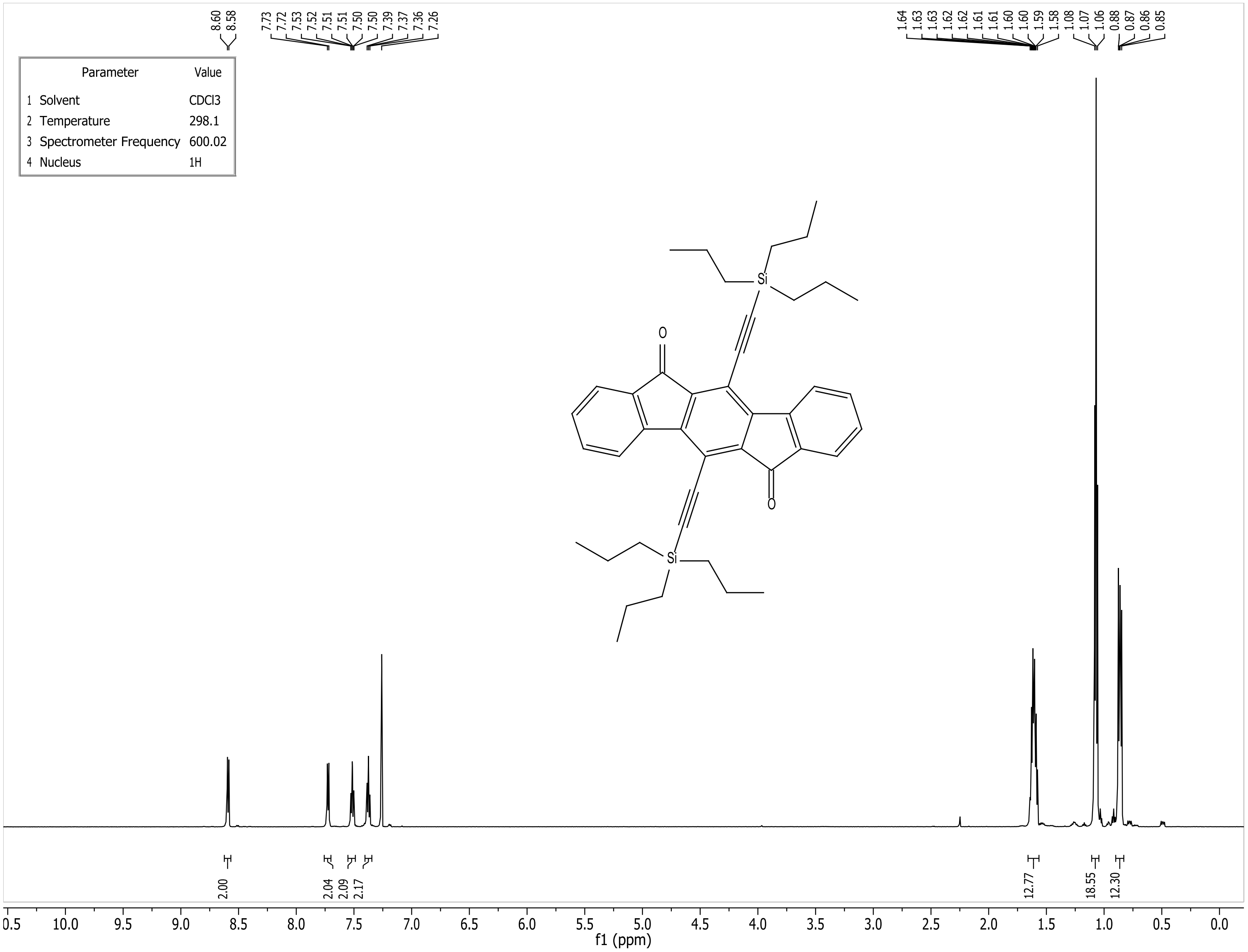

8.60
8.58
7.73
7.72
7.53
7.52
7.51
7.51
7.50
7.50
7.39
7.37
7.36
7.26
1.64
1.63
1.63
1.62
1.62
1.61
1.61
1.60
1.60
1.59
1.58
1.08
1.07
1.06
0.88
0.87
0.86
0.85
Parameter Value
1 Solvent CDCl3
2 Temperature 298.1
3 Spectrometer Frequency 600.02
4 Nucleus 1H
O
O
Si
Si
2.00
2.04
2.09
2.17
12.77
18.55
12.30
f1 (ppm)

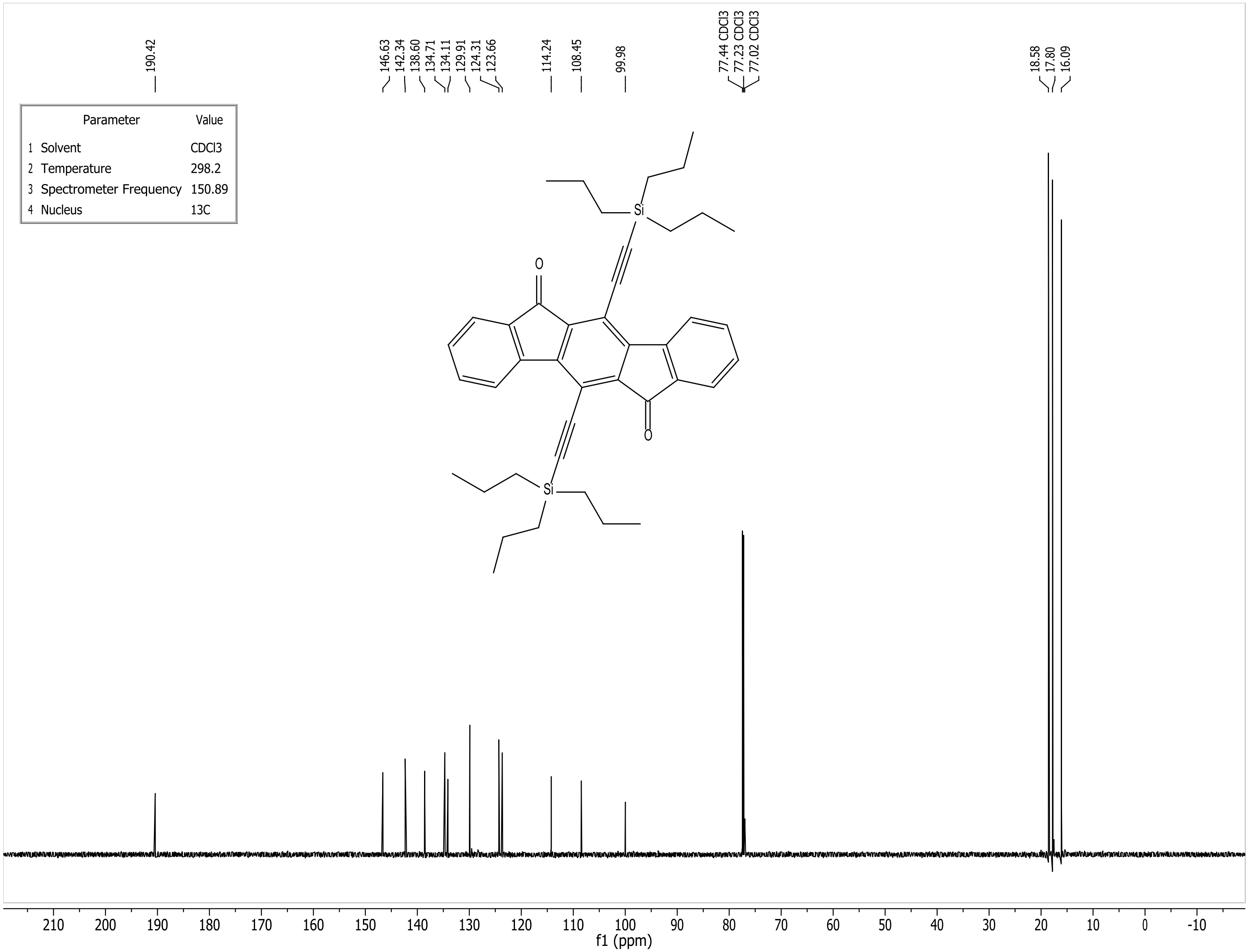

190.42
146.63
142.34
138.60
134.71
134.11
129.91
124.31
123.66
114.24
108.45
99.98
77.44 CDCl3
77.23 CDCl3
77.02 CDCl3
18.58
17.80
16.09
Parameter Value
1 Solvent CDCl3
2 Temperature 298.2
3 Spectrometer Frequency 150.89
4 Nucleus 13C
O
Si
f1 (ppm)
210 200 190 180 170 160 150 140 130 120 110 100 90 80 70 60 50 40 30 20 10 0 -10

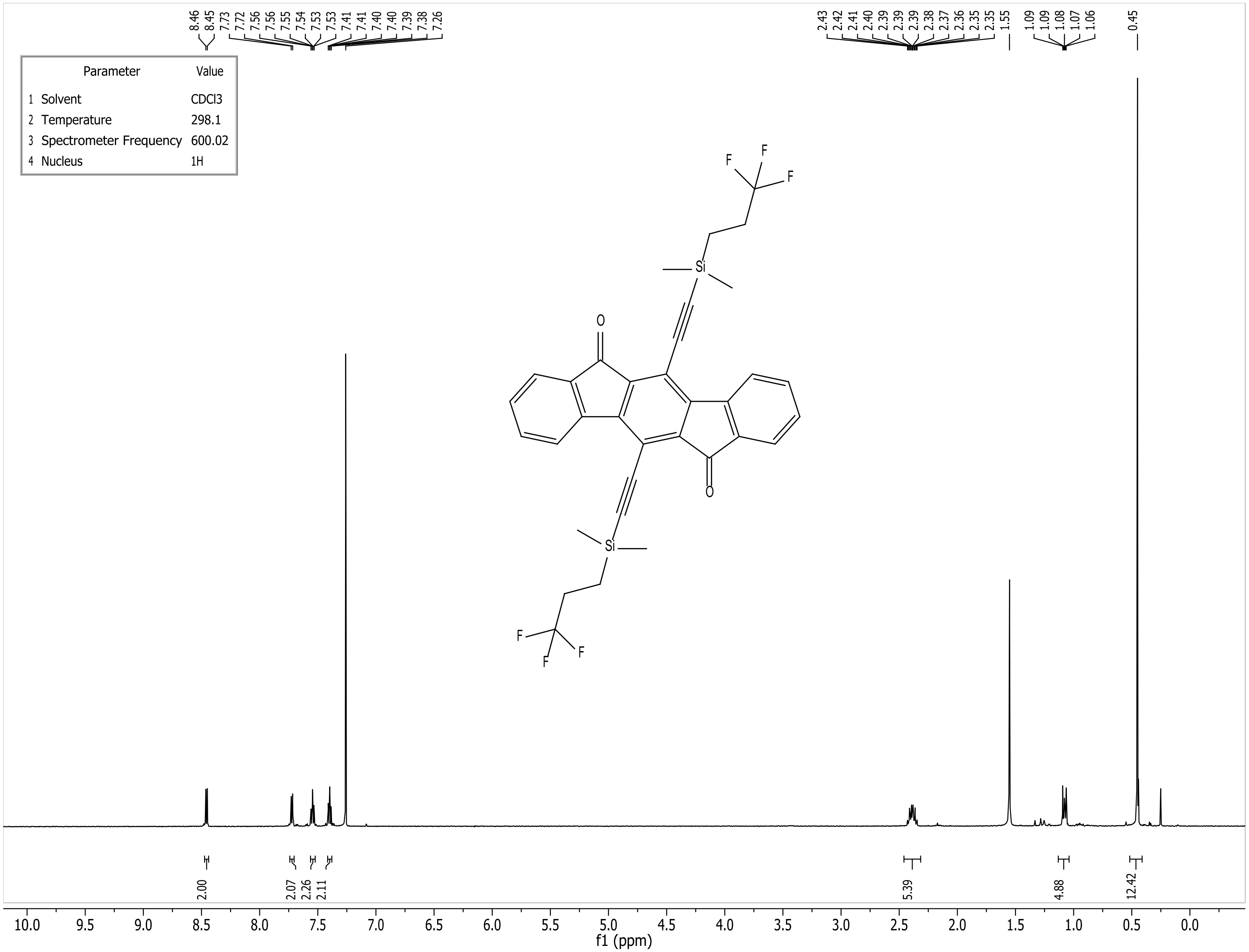
8.46
8.45
7.73
7.72
7.56
7.56
7.55
7.54
7.53
7.53
7.41
7.41
7.40
7.39
7.38
7.26
2.43
2.42
2.41
2.40
2.39
2.39
2.39
2.38
2.37
2.36
2.35
2.35
1.55
1.09
1.09
1.08
1.07
1.06
0.45
Parameter
Value
1 Solvent
CDCl3
2 Temperature
298.1
3 Spectrometer Frequency
600.02
4 Nucleus
1H
F
F
F
Si
O
O
Si
F
F
F
2.00
2.07
2.26
2.11
5.39
4.88
12.42
10.0
9.5
9.0
8.5
8.0
7.5
7.0
6.5
6.0
5.5
5.0
4.5
4.0
3.5
3.0
2.5
2.0
1.5
1.0
0.5
0.0
f1 (ppm)

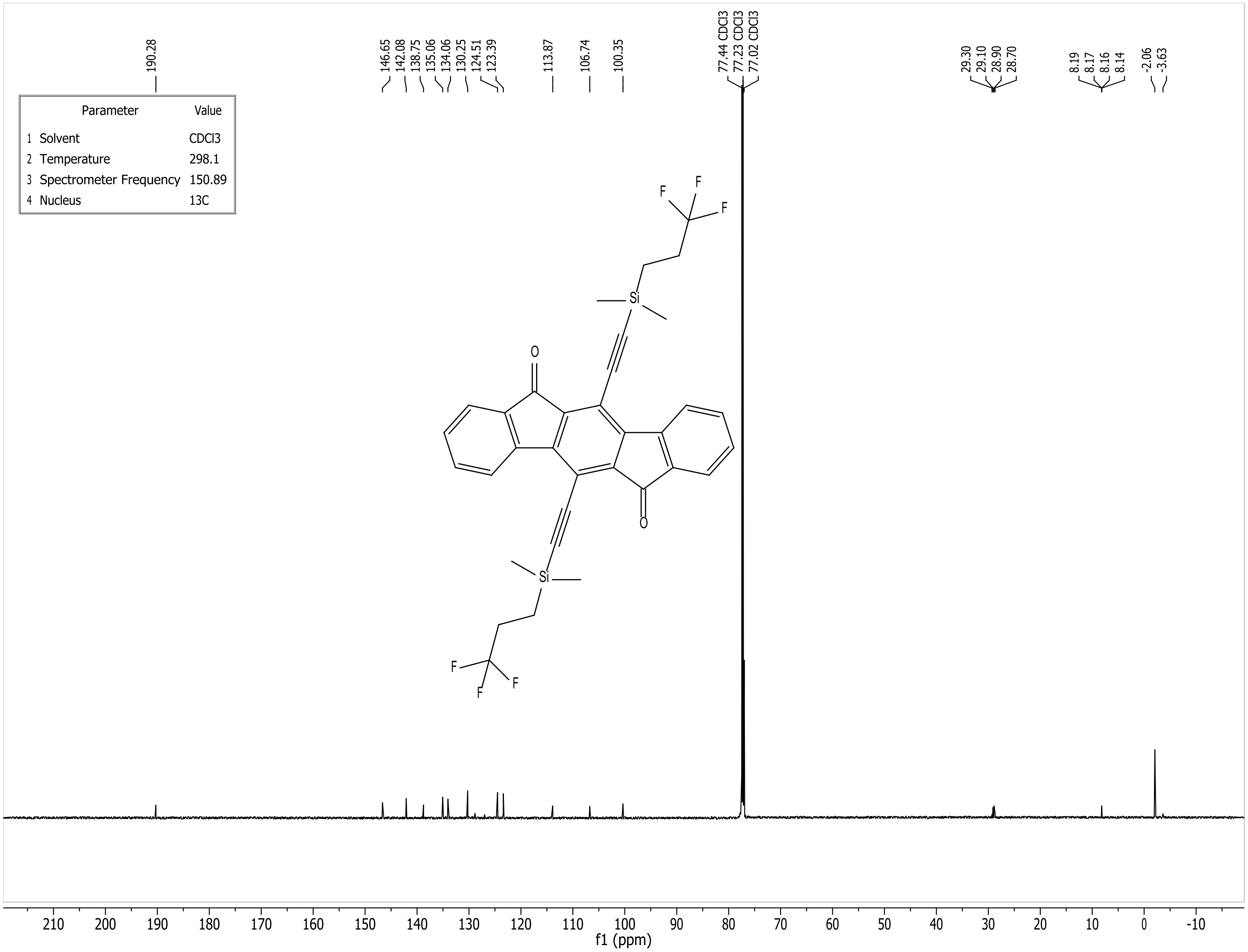

190.28
146.65
142.08
138.75
135.06
134.06
130.25
124.51
123.39
113.87
106.74
100.35
77.44 CDCl3
77.23 CDCl3
77.02 CDCl3
29.30
29.10
28.90
28.70
8.19
8.17
8.16
8.14
-2.06
-3.63
Parameter Value
1 Solvent CDCl3
2 Temperature 298.1
3 Spectrometer Frequency 150.89
4 Nucleus 13C
O
O
Si
Si
F F F
F F F
210 200 190 180 170 160 150 140 130 120 110 100 90 80 70 60 50 40 30 20 10 0 -10
f1 (ppm)

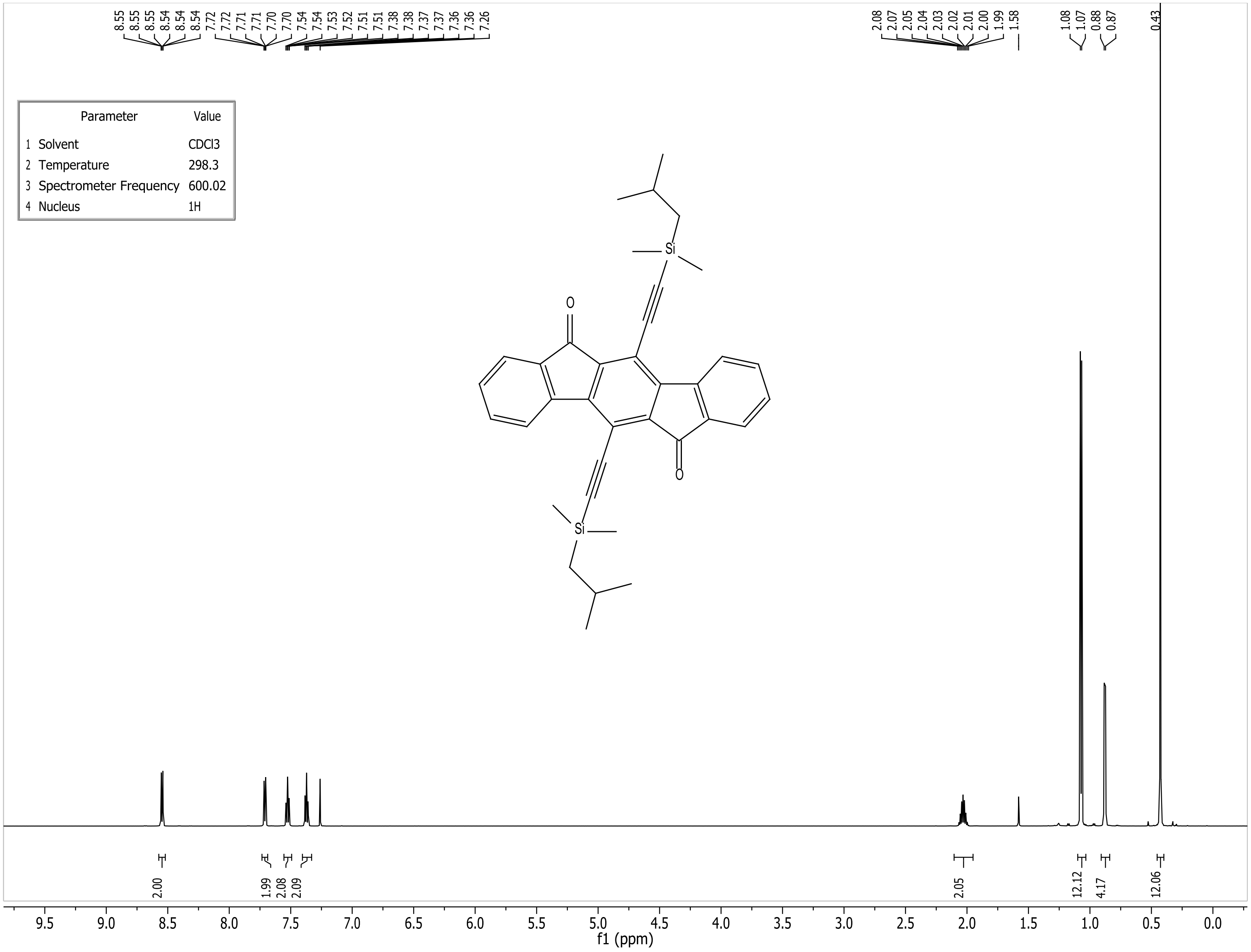

8.55
8.55
8.55
8.54
8.54
8.54
7.72
7.72
7.71
7.71
7.70
7.70
7.54
7.54
7.53
7.52
7.51
7.51
7.38
7.38
7.37
7.37
7.36
7.36
7.26
2.08
2.07
2.05
2.04
2.03
2.02
2.01
2.00
1.99
1.58
1.08
1.07
0.88
0.87
0.43
Parameter
Value
1 Solvent
CDCl3
2 Temperature
298.3
3 Spectrometer Frequency
600.02
4 Nucleus
1H
O
O
Si
Si
2.00
1.99
2.08
2.09
2.05
12.12
4.17
12.06
9.5
9.0
8.5
8.0
7.5
7.0
6.5
6.0
5.5
5.0
4.5
4.0
3.5
3.0
2.5
2.0
1.5
1.0
0.5
0.0
f1 (ppm)

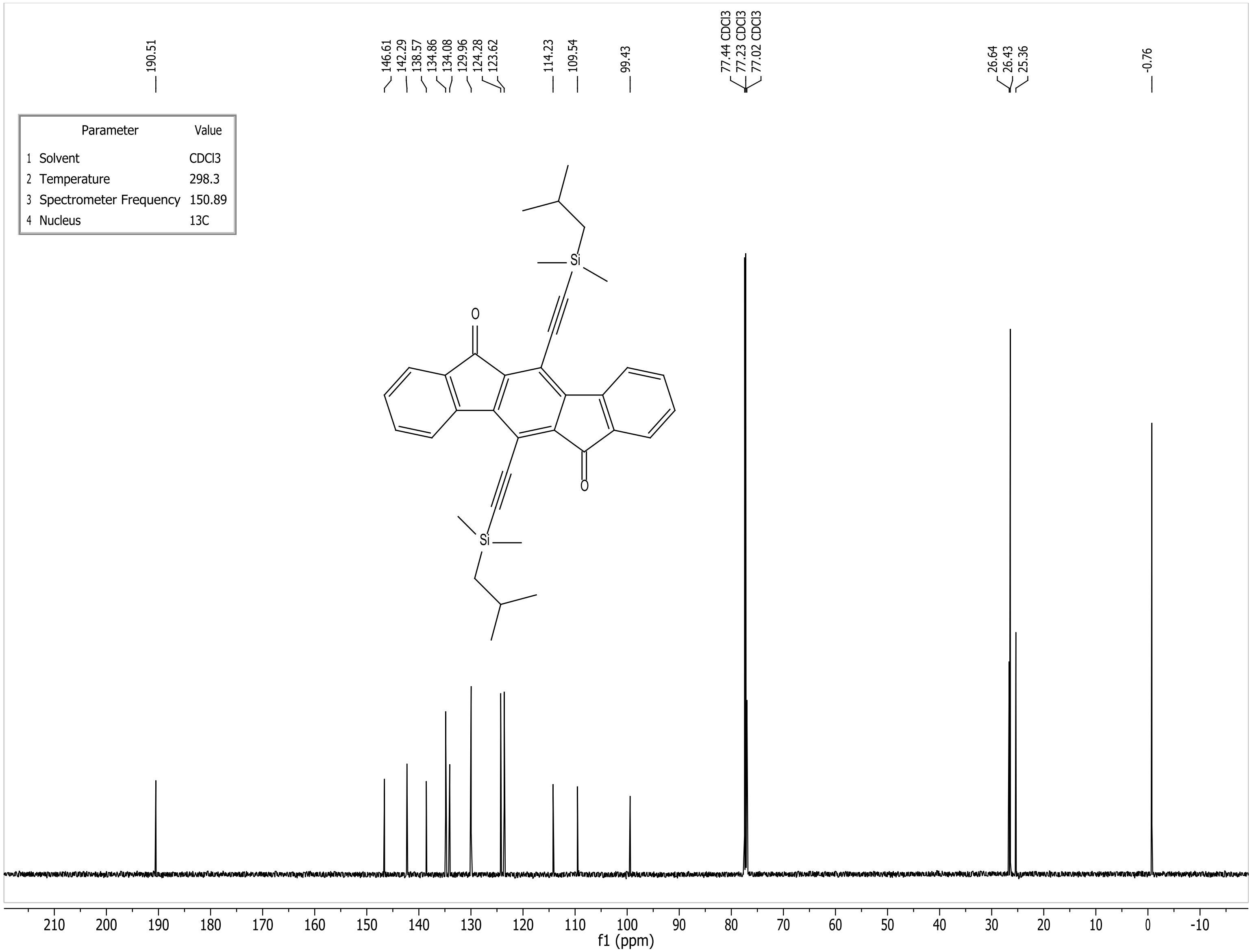
190.51
146.61
142.29
138.57
134.86
134.08
129.96
124.28
123.62
114.23
109.54
99.43
77.44 CDCl3
77.23 CDCl3
77.02 CDCl3
26.64
26.43
25.36
-0.76
Parameter Value
1 Solvent CDCl3
2 Temperature 298.3
3 Spectrometer Frequency 150.89
4 Nucleus 13C
O
Si
f1 (ppm)
210 200 190 180 170 160 150 140 130 120 110 100 90 80 70 60 50 40 30 20 10 0 -10

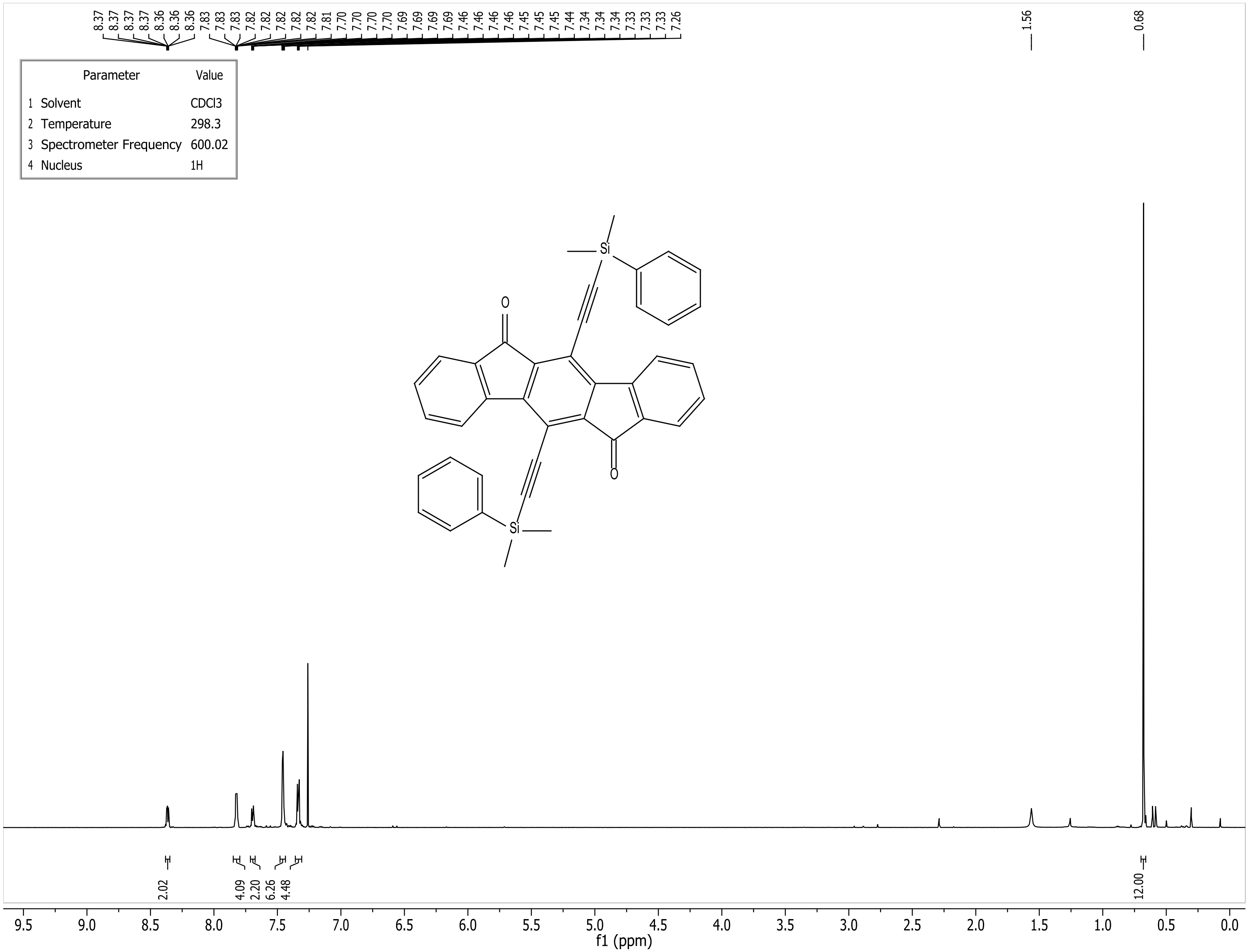
8.37
8.37
8.37
8.37
8.36
8.36
8.36
7.83
7.83
7.82
7.82
7.82
7.82
7.82
7.81
7.70
7.70
7.70
7.70
7.69
7.69
7.69
7.69
7.46
7.46
7.46
7.45
7.45
7.45
7.44
7.34
7.34
7.34
7.33
7.33
7.33
7.26
1.56
0.68
Parameter
Value
1 Solvent
CDCl3
2 Temperature
298.3
3 Spectrometer Frequency
600.02
4 Nucleus
1H
O
Si
O
Si
2.02
4.09
2.20
6.26
4.48
12.00
f1 (ppm)

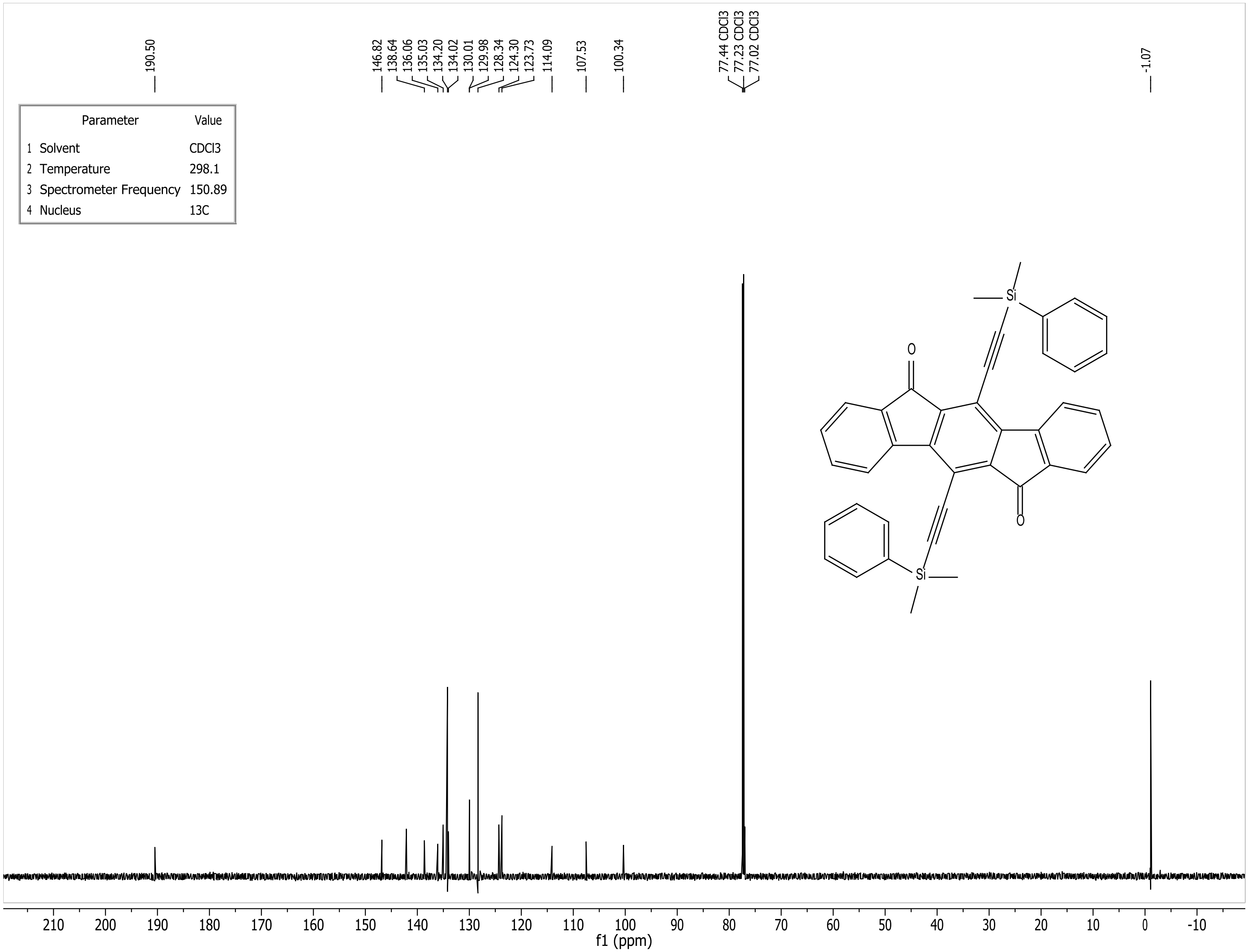
190.50
146.82
138.64
136.06
135.03
134.20
134.02
130.01
129.98
128.34
124.30
123.73
114.09
107.53
100.34
77.44 CDCl3
77.23 CDCl3
77.02 CDCl3
-1.07
Parameter Value
1 Solvent CDCl3
2 Temperature 298.1
3 Spectrometer Frequency 150.89
4 Nucleus 13C
O
O
Si
Si
210 200 190 180 170 160 150 140 130 120 110 100 90 80 70 60 50 40 30 20 10 0 -10
f1 (ppm)
